# Comparative efficacy of Chinese herbal injections combined with azithromycin for mycoplasma pneumonia in children: A Bayesian network meta‐analysis of randomized controlled trials

**DOI:** 10.1111/jcpt.12855

**Published:** 2019-05-22

**Authors:** Xiaojiao Duan, Kaihuan Wang, Jiarui Wu, Dan Zhang, Xinkui Liu, Mengwei Ni, Shuyu Liu, Ziqi Meng

**Affiliations:** ^1^ Department of Clinical Chinese Pharmacy, School of Chinese Materia Medica Beijing University of Chinese Medicine Beijing China

**Keywords:** azithromycin, Chinese herbal injection, *Mycoplasma pneumonia*, mycoplasma pneumonia in children, network meta‐analysis, systematic review

## Abstract

**What is known and objective:**

An increasing macrolide resistance leads to complex clinical treatment schemes in mycoplasma pneumonia in children. Chinese herbal injection (CHI) is widely used to treat it and may provide a new treatment regimen. This study was conducted to systematically evaluate the efficacy of CHIs combined with azithromycin for treating mycoplasma pneumonia in children by Bayesian network meta‐analysis.

**Methods:**

Randomized controlled trials (RCTs) of CHIs combined with azithromycin for mycoplasma pneumonia in children were searched in electronic databases and related references from initiation to 30 October 2018. Two researchers conducted data extraction and risk of bias assessment. WinBUGS software and STATA software were adopted to analyse the data.

**Results:**

A total of 167 RCTs were included with 5 CHIs involving 16 144 patients. All CHIs combined with azithromycin had superior effects than azithromycin only among overall outcomes. Yanhuning injection combined with azithromycin ranked highest in four different outcomes and second in two based on surface under the cumulative ranking probabilities (SUCRA). Meanwhile, the results of MD and 95% CIs of concerned outcomes indicated that only Yanhuning injection combined with azithromycin had better response than other CHIs combined with azithromycin. Moreover, cluster analysis results revealed Reduning injection combined with azithromycin was associated with a positive effect on the three group outcomes. Similarly, it was found to be the top three ranking in all outcomes based on SUCRA.

**What is new and conclusion:**

Yanhuning injection combined with azithromycin and Reduning injection combined with azithromycin were found to be preferable treatments based on the data of this study.

## 1 WHAT IS KNOWN AND OBJECTIVE

*Mycoplasma pneumoniae* is an atypical bacterium that can cause severe respiratory tract infections.^1^, ^2^ In addition, it is a significant cause leading to the hospitalization of children with community‐acquired pneumonia.^3^, ^4^ The incidence of mycoplasma pneumonia in children is high, which accounts for 16%‐30% of community‐acquired pneumonia in children.^5^, ^6^, ^7^, ^8^ It is generally mild, but it can be very serious, even involves extra pulmonary spread, such as central nervous system, mucosa and other organs, which seriously endangered the health of children.^4^ Due to the serious side effects of tetracycline and fluoroquinolones on children, macrolides have become first choice for the treatment of mycoplasma pneumonia in children.^3^, ^9^ Azithromycin, a macrolide antibiotic, is a preferred decision because of its long half‐life and clear target‐cell effect.^10^ However, macrolide resistance has emerged worldwide with a significant increase, which increasingly lead to complex clinical treatment schemes.^2^, ^11^, ^12^, ^13^, ^14^, ^15^ In recent years, with the increase of people’ recognition of traditional Chinese medicine, combining Chinese Medicine and Western Medicine to treat diseases has been an acceptable treatment method.^16^, ^17^ This provides another idea to treat mycoplasma pneumonia in children.

Chinese herbal injection (CHI) is a new formulation with rapid action and high bioavailability and made from components extracted from traditional Chinese herb using modern technology.^18^ It is widely used in the treatment of cardiovascular diseases, cancers and respiratory diseases.^19^, ^20^, ^21^, ^22^, ^23^, ^24^ Although numerous traditional pairwise meta‐analytic reviews have reported on the efficacy of many CHIs that could be used to treat mycoplasma pneumonia in children,^25^, ^26^, ^27^, ^28^ the effect of which kind of injection is the best remains unclear. This is because the traditional pairwise meta‐analysis can only achieve direct comparison between the two interventions and cannot comprehensively evaluate the efficacy of various interventions. Network meta‐analysis is an extension of traditional pairwise meta‐analysis to multiple treatment comparisons and is a good choice when there is a lack of direct evidence, because it allows indirect comparisons between different types of treatment.^29^, ^30^

Therefore, this study used Bayesian network meta‐analysis to systematically evaluate the efficacy of CHIs for the treatment of mycoplasma pneumonia in children and obtain the ranking of their effect in different outcomes, to provide evidence of evidence‐based medicine for the clinicians to choose a more suitable therapy.

## 2 METHODS

**Figure 1:**
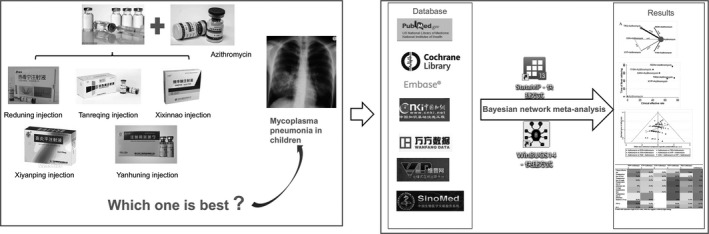
Design idea and concise workflow

The study was conducted according to the PRISMA extension statement (Appendix S1).^31^ In addition, the design idea and concise workflow of this study are presented in Figure 1.

### 2.1 Eligibility criteria

Eligibility criteria were formulated in accordance with PICOS checklist.^32^ *Population*. Patients were under 15 years old and received definitive diagnosis of mycoplasma pneumonia. *Intervention and Control*. After the preanalysis, the following interventions were included: Azithromycin, Reduning injection combined with azithromycin, Tanreqing injection combined with azithromycin, Xixinnao injection combined with azithromycin, Xiyanping injection combined with azithromycin, Yanhuning injection combined with azithromycin. More details about the product information of 5 CHIs are presented in Appendix S2 including raw materials, component ingredients to be measured, botanical plant names, labelled efficacy, indications, standard of authority and adverse drug reactions. Basic therapies were given if necessary, including abatement of fever, relieving a cough, preventing asthma, reducing phlegm. *Outcomes*. The following outcomes attracted our attention: clinical effective rate, disappearance time of fever, disappearance time of cough, disappearance time of pulmonary rale, average hospitalization time, disappearance time of pulmonary shadows in X‐ray, serum level of TNF‐α and IL‐6. Clinical effective rate = (number of total patients – number of invalid patients)/number of total patients × 100%. The patients whose clinical symptoms were unchanged and objective indicators were not adjusted were deemed as invalid. *Study design*. Only randomized controlled trials (RCTs) were enrolled.

However, RCTs that lacked course of treatment and correct generation of random sequence were excluded.

### 2.2 Search strategy

RCTs were identified through China National Knowledge Infrastructure Database, Wan‐Fang Database, Chinese Scientific Journals Full‐text Database, Chinese Biomedical Literature Database, PubMed, Cochrane Library and Embase from inception to 30 October 2018. In addition, the references of relevant studies were confirmed. The detail of search terms is showed in Appendix S3.

### 2.3 Data extraction and risk of bias assessment

Two researchers (XD and DZ) decided included RCTs independently on the basis of eligibility criteria. All identified literatures were arranged by NoteExpress software (Wuhan University Library). The information and data of enrolled RCTs were organized via Microsoft Excel 2016 software including name of first author, published year, the number of gender and age of patients, details of interventions, measured data of outcomes and factors to evaluate risk of bias. Items of risk of bias consisted of generation of random sequence, allocation concealment, blinding of participants and personnel, blinding of outcome assessment, incomplete outcome data, selective reporting and other bias. Two researchers (XD and ZM) assessed risk of bias independently according to Cochrane Risk of Bias Tool.^33^ Each item had three levels: low risk, unclear and high risk. Discordance of two researchers resolved by consensus or a third opinion.

Ethical approval was unnecessary, as private information of patients was not involved in this study.

### 2.4 Statistical analysis

Data analysis was conducted by WinBUGS 1.4.3 software (MRC Biostatistics Unit) and STATA 13.0 software (Stata Corporation) based on the Bayesian hierarchical model and Markov Chain Monte Carlo algorithm. Clinical effective rate was analysed using odds ratio (OR) with 95% confidence intervals (CIs), and mean difference (MD) with 95% CIs was conducted to explain other outcomes. All of them were generated via random effects model. There was no statistically significant difference between groups when OR's 95% CIs did not contain one or MD's 95% CIs did not contain zero. A total of 200 000 iterations were set up for calculation, and the first 10 000 of them ran for burn‐in to remove the influence of original value. Surface under the cumulative ranking probabilities (SUCRA) was computed to estimate the treatment hierarchy of targeted interventions. Higher SUCRA symbolized better effect. Network plot was drawn to illustrate the relationship of included treatments. Publication bias was identified by comparison‐adjusted funnel plot based on the data of clinical effective rate. We implemented the clustering analysis based on SUCRA to assess comprehensive efficacy of competing treatments in any two outcomes.

## 3 RESULTS

### 3.1 Literature retrieval and study characteristics

**Figure 2:**
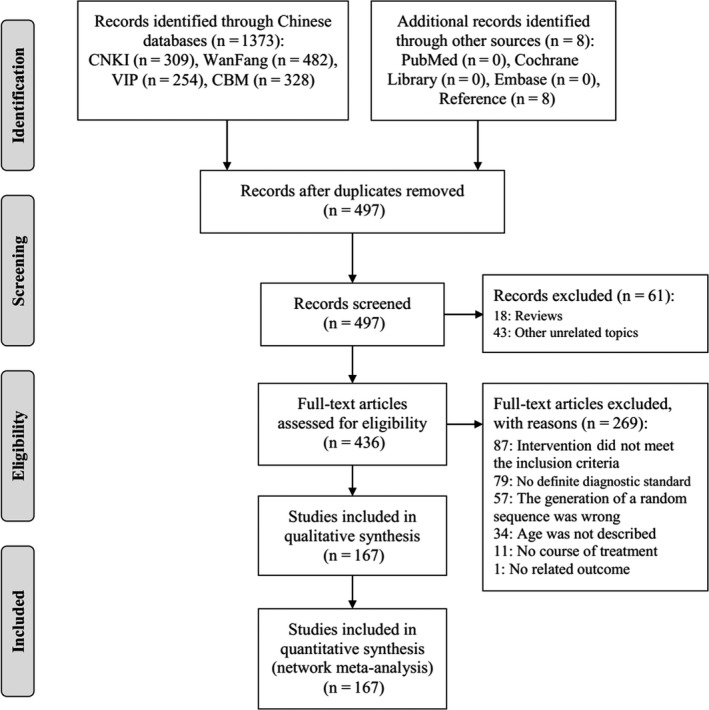
Flow diagram of study search. CBM, Chinese Biomedical Literature Database; CNKI, China National Knowledge Infrastructure Database; n, number of articles; VIP, the Chinese Scientific Journals Full‐text Database; WanFang, Wan‐Fang Database

**Figure 3:**
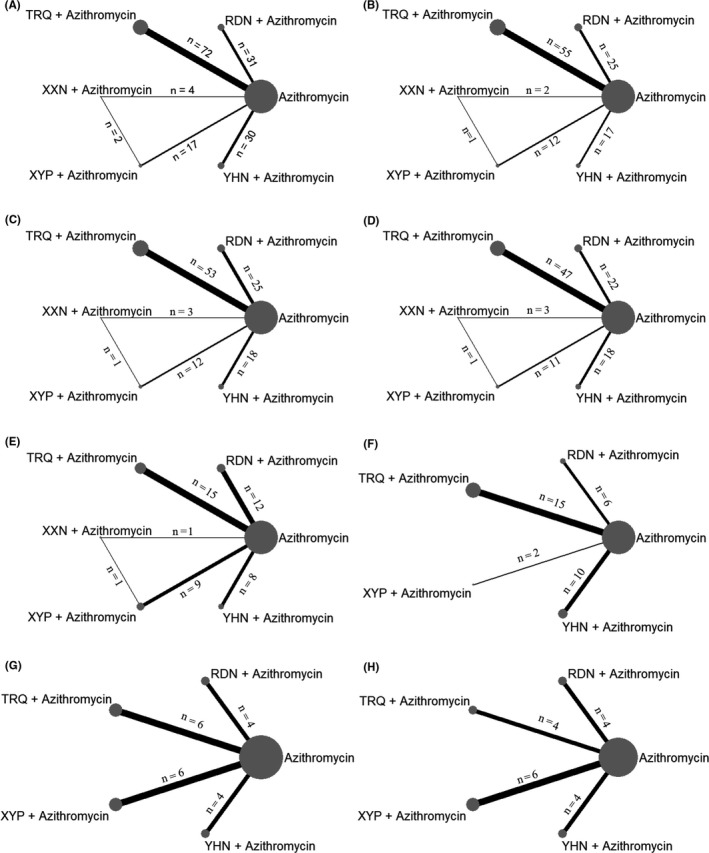
Network plot for different outcomes. A, clinical effective rate; B, disappearance time of fever; C, disappearance time of cough; D, disappearance time of pulmonary rale; E, average hospitalization time; F, disappearance time of pulmonary shadows in X‐ray; G, TNF‐α; H, IL‐6; RDN, Reduning injection. n, the number of RCTs; TRQ, Tanreqing injection; XXN, Xixinnao injection; XYP, Xiyanping injection; YHN, Yanhuning injection

A total of 1181 articles were identified at first, and 167 RCTs were remained after rigorous screening. The procedure of screening is shown in Figure 2. All RCTs were conducted in China and published between 2005 and 2018. Figure 3 and Appendix S4 present the relationship of competing interventions with different outcomes and detail of included RCTs, respectively. A total of 16 144 patients were reported in 167 RCTs. Patients who were given only azithromycin were 7909, Reduning injection combined with azithromycin were 1480, Tanreqing injection combined with azithromycin were 3635, Xixinnao injection combined with azithromycin were 242, Xiyanping injection combined with azithromycin were 1054 and Yanhuning injection combined with azithromycin were 1824. Maximum sample size was 167, and minimum was 18. A total of 156 RCTs (93.41%) reported clinical effective rate. Hundred and twelve RCTs (67.07%), 112 RCTs (67.07%), 102 RCTs (61.08%), 46 RCTs (27.54%), 33 RCTs (19.76%), 20 RCTs (11.97%) and 18 RCTs (10.78%) reported disappearance time of fever, disappearance time of cough, disappearance time of pulmonary rale, average hospitalization time, disappearance time of pulmonary shadows in X‐ray, TNF‐α and IL‐6, respectively.

### 3.2 Risk of bias assessment

**Figure 4:**
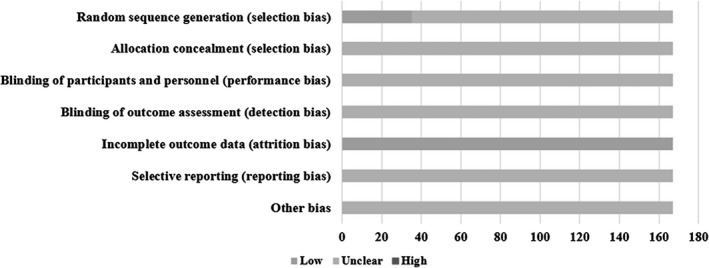
Assessment of risk bias

A total of 34 RCTs generated random sequence through random number table and 1 RCT through coin tossing. Therefore, their selection bias of random sequence generation was regarded as low risk. Attrition bias of incomplete outcome data was assessed as low risk due to all the research data was complete. The remaining biases were estimated as unclear because there were too few details could obtain to make a decision. In short, the quality of eligible RCTs was general (Figure 4).

### 3.3 Results of overall outcomes

The result data of all outcomes are presented in Table 1. As for clinical effective rate, all CHIs combined with azithromycin were superior to azithromycin only. When compared with azithromycin, all treatments, except Xixinnao injection combined with azithromycin, provided significant benefits in reducing disappearance time of fever, cough and pulmonary rale, and average hospitalization time. Furthermore, Yanhuning injection combined with azithromycin was found to be more efficacious than Xiyanping injection combined with azithromycin in decreasing disappearance time of cough and average hospitalization time, and other CHIs combined with azithromycin in lessening disappearance time of pulmonary rale. In terms of disappearance time of pulmonary shadows in X‐ray, Reduning injection combined with azithromycin, Xiyanping injection combined with azithromycin and Yanhuning injection combined with azithromycin yielded significant effects than azithromycin alone. As for concentration of TNF‐α and IL‐6, only Reduning injection combined with azithromycin could achieve a better efficacy than azithromycin. The significant difference was merely observed between above groups.

**Table 1:** Network meta‐analysis results of OR and MD with 95%CIs for eight outcomes

	Clinical effective rate	Disappearance time of cough fever	Disappearance time of cough	Disappearance time of pulmonary rale	Average hospitalization time	Disappearance time of pulmonary shadows in X‐ray	TNF‐α	IL‐6
RDN + Azithromycin vs.								
XYP + Azithromycin	0.94 (0.58, 1.51)	−0.43 (−1.36, 0.52)	−0.61 (−1.89, 0.6)	−0.52 (−2.02, 0.81)	−0.76 (−2.61, 1.13)	0.92 (−1.97, 3.76)	−2.04 (−8.71, 5.56)	−0.2 (−7.71, 7.34)
XXN + Azithromycin	0.84 (0.41, 1.84)	−0.19 (−2.02, 1.59)	−0.5 (−2.8, 1.55)	−0.52 (−2.44, 1.24)	−0.85 (−4.9, 2.91)	–	–	–
YHN + Azithromycin	0.82 (0.55, 1.21)	−0.14 (−1, 0.77)	0.49 (−0.55, 1.51)	**1.87 (0.8, 2.86)**	0.99 (−1.13, 2.99)	1.59 (−0.4, 3.56)	−2.83 (−8.55, 2.94)	−0.14 (−6.58, 5.78)
TRQ + Azithromycin	1.01 (0.70, 1.43)	−0.38 (−1.16, 0.43)	0.34 (−0.83, 1.49)	0.1 (−1.13, 1.28)	0.22 (−1.81, 2.14)	−0.23 (−3.01, 2.34)	−1.78 (−6.95, 3.89)	−3.05 (−9.11, 3.16)
Azithromycin	**0.16 (0.12, 0.22)**	−**1.63 (−2.32, **−**0.95)**	−**2.23 (−3.15, **−**1.31)**	−**1.96 (−2.97, **−**0.96)**	−**2.26 (−3.87, **−**0.81)**	−**2.18 (−4.09, **−**0.29)**	−**3.71 (−7.69, **−**0.16)**	−**4.09 (−7.8, **−**0.59)**
XYP + Azithromycin vs.								
XXN + Azithromycin	0.88 (0.47, 1.85)	0.23 (−1.49, 1.95)	0.08 (−1.86, 2.06)	0 (−1.25, 1.18)	−0.18 (−3.91, 3.43)	–	–	–
YHN + Azithromycin	0.87 (0.56, 1.35)	0.29 (−0.54, 1.18)	**1.1 (0.19, 2.03)**	**2.41 (1.4, 3.33)**	**1.71 (0.02, 3.45)**	0.7 (−1.54, 2.92)	−0.77 (−8.65, 6.17)	0.03 (−8.59, 8.31)
TRQ + Azithromycin	1.07 (0.69, 1.60)	0.05 (−0.69, 0.85)	0.96 (−0.07, 1.94)	0.64 (−0.54, 1.73)	0.95 (−0.65, 2.58)	−1.16 (−4, 1.68)	0.25 (−7.07, 7.14)	−2.83 (−11.2, 5.44)
Azithromycin	**0.17 (0.12, 0.25)**	−**1.21 (−1.85, **−**0.55)**	−**1.63 (−2.43, **−**0.79)**	−**1.42 (−2.4, **−**0.51)**	−**1.55 (−2.65, **−**0.45)**	−**3.07 (−5.26, **−**0.92)**	−1.63 (−8.47, 3.72)	−3.89 (−10.6, 2.6)
XXN + Azithromycin vs.								
YHN + Azithromycin	0.98 (0.46, 2.21)	0.07 (−1.64, 1.84)	1.01 (−0.91, 2.98)	**2.44 (0.92, 3.93)**	1.88 (−2, 5.71)	–	–	–
TRQ + Azithromycin	1.20 (0.57, 2.41)	−0.18 (−1.84, 1.58)	0.88 (−1.11, 2.86)	0.65 (−0.95, 2.28)	1.14 (−2.76, 5.07)	–	–	–
Azithromycin	**0.20 (0.10, 0.38)**	−1.44 (−3.09, 0.27)	−1.71 (−3.56, 0.23)	−1.39 (−2.87, 0.11)	−1.39 (−5.02, 2.35)	–	–	–
YHN + Azithromycin vs.								
TRQ + Azithromycin	1.23 (0.89, 1.72)	−0.24 (−0.91, 0.4)	−0.14 (−0.97, 0.65)	−**1.77 (−2.54**, −**1.06)**	−0.73 (−2.65, 1.06)	−1.87 (−3.85, 0.13)	1.03 (−4.49, 7.09)	−2.89 (−9.89, 4.56)
Azithromycin	**0.20 (0.15**, **0.26)**	−**1.49 (−2.1**, −**0.99)**	−**2.72 (−3.19**, −**2.26)**	−**3.84 (−4.07**, −**3.5)**	−**3.25 (−4.62**, −**1.92)**	−**3.77 (−4.3**, −**3.26)**	−0.84 (−5.51, 3.24)	−3.95 (−8.82, 1.23)
TRQ + Azithromycin vs.								
Azithromycin	**0.16 (0.14**, **0.20)**	−**1.26 (−1.68**, −**0.89)**	−**2.57 (−3.21**, −**1.89)**	−**2.07 (−2.69**, −**1.33)**	−**2.48 (−3.72**, −**1.27)**	−1.9 (−3.86, 0)	−1.94 (−6.32, 1.58)	−1.05 (−6.22, 3.9)

Note:

Highlighted results mean there are statistically significant differences between two groups.

Abbreviations: RDN, Reduning injection; TRQ, Tanreqing injection; XXN, Xixinnao injection; XYP, Xiyanping injection; YHN, Yanhuning injection.

### 3.4 Ranking results based on SUCRA

SUCRA results of eight outcomes are depicted in Table 2. Azithromycin was the worst treatment in all outcomes. Tanreqing injection combined with azithromycin had a 74.6% probability to be the best intervention measure for clinical effective rate, successively followed by Reduning injection combined with azithromycin (SUCRA: 71.6%) and Xiyanping injection combined with azithromycin (SUCRA: 63.3%). The top three therapies for disappearance time of fever were Reduning injection combined with azithromycin (SUCRA: 76.8%), Yanhuning injection combined with azithromycin (SUCRA: 68.7%) and Xixinnao injection combined with azithromycin (SUCRA: 60.3%). Yanhuning injection combined with azithromycin ranked highest in terms of disappearance time of cough, pulmonary rale and pulmonary shadows in X‐ray, and average hospitalization time (SUCRA: 85.5%, 100%, 91% and 88.4%, respectively). Similarly, Reduning injection combined with azithromycin ranked third for above four outcomes. As for the second, average hospitalization time, disappearance time of cough and pulmonary rale were Tanreqing injection combined with azithromycin (SUCRA: 61%, 59.9% and 59%, respectively), and disappearance time of pulmonary shadows in X‐ray was Xiyanping injection combined with azithromycin (SUCRA: 69.8%). In terms of concentration of TNF‐α and IL‐6, Reduning injection combined with azithromycin (SUCRA: 83.2%, 71.7%) was the most like to be the best therapy followed by Tanreqing injection combined with azithromycin (SUCRA: 57.2%) and Yanhuning injection combined with azithromycin (SUCRA: 67.9%), respectively.

**Table 2:**
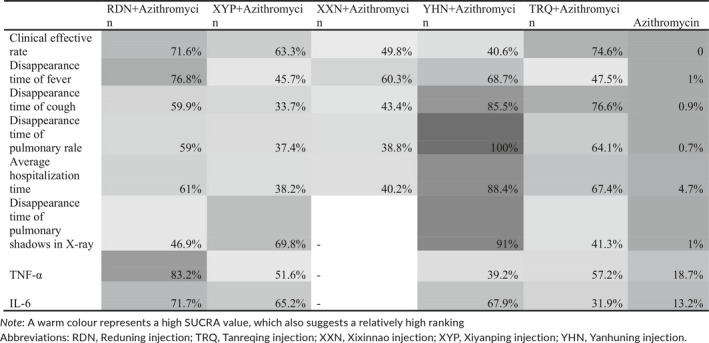
Surface under the cumulative ranking probabilities (SUCRA) results of eight outcomes

### 3.5 Cluster analysis

**Figure 5:**
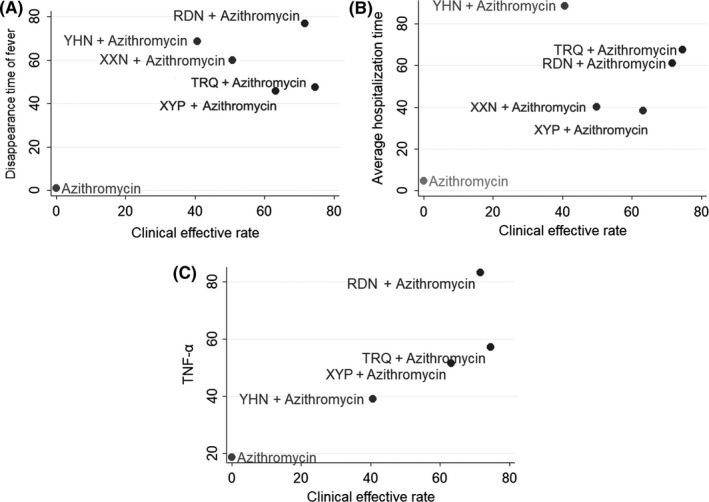
Cluster analysis plot for four outcomes. Interventions with the same colour belonged to the same cluster, and interventions located in the upper right corner indicate optimal therapy for two different outcomes

Three groups of cluster analysis were performed in this study, including clinical effective rate and disappearance time of fever, clinical effective rate and average hospitalization time, clinical effective rate and TNF‐α. The results are presented in Figure 5. Through comprehensive analysis based on cluster analysis, Reduning injection combined with azithromycin was associated with preferable response in clinical effective rate and disappearance time of fever, clinical effective rate and TNF‐α. Moreover, when combined with azithromycin, Reduning injection and Tanreqing injection showed a favourable improvement in clinical effective rate and average hospitalization time.

### 3.6 Publication bias

**Figure 6:**
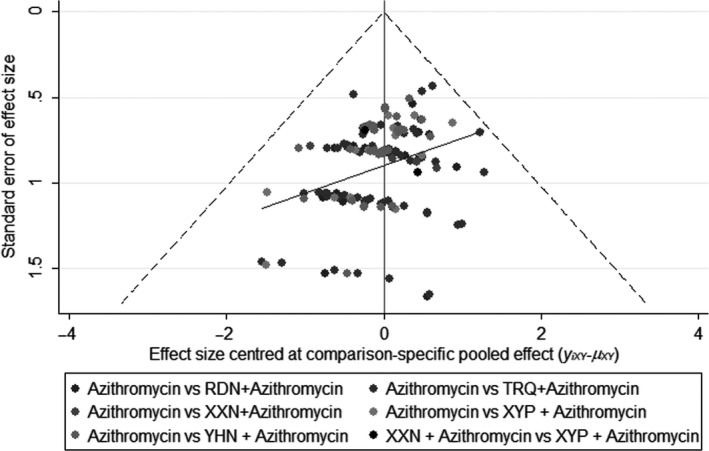
Comparison‐adjusted funnel plot for the rate of clinical efficacy.Points with different colours represent different interventions. If the points distributed in the funnel are symmetrical, there is no publication bias

Comparison‐adjusted funnel plot for clinical effective rate was drawn to test the publication bias. Figure 6 shows that the points located in the funnel plot were asymmetrical based on the zero line, and the angle between the adjusted auxiliary line and the zero line was larger. Therefore, there may be a small publication bias.

### 3.7 Safety

Among the all of 167 RCTs, 95 RCTs (56.89%) reported adverse drug reactions (ADRs) during the course of treatment. Nine out of them declared there was not ADRs, and 86 out of them described the detail of ADRs. The incidence of different types of ADRs in different interventions is counted in Table 3. The remaining 72 (43.11%) did not monitor ADRs during treatment. We could reach two main points from Table 3: first, patients with azithromycin alone had the highest incidence of ADRs; second, the incidence of gastrointestinal reactions was the highest among all competing interventions.

**Table 3:** The incidence of different types of adverse drug reactions in different interventions

	RDN + azithromycin	XYP + azithromycin	XXN + azithromycin	YHN + azithromycin	TRQ + azithromycin	Azithromycin
Gastrointestinal reactions	6.71% (46/686)	5.96% (41/688)	9.38% (6/64)	7.33% (55/750)	7.25% (144/1986)	14.26% (573/4018)
Skin rash	2.19% (15/686)	2.03% (14/688)	6.25% (4/64)	0.13% (1/750)	0.30% (6/1986)	1.07% (43/4018)
Dizziness	0.15% (1/686)	0.29% (2/688)	–	0.27% (2/750)	0.15% (3/1986)	0.17% (7/4018)
Liver dysfunction	1.60% (11/686)	0.44% (3/688)	–	–	0.10% (2/1986)	0.52% (21/4018)
Pain at the injection site	–	1.74% (12/688)	–	1.47% (11/750)	0.20% (4/1986)	0.77% (31/4018)
Aseptic meningitis	0.15% (1/686)	–	–	–	–	–
Reduction of leucocyte	–	–	–	–	–	0.07% (3/4018)
Phlebitis	–	–	–	–	–	0.12% (5/4018)
Limbs weakness	–	–	–	–	–	0.05% (2/4018)
Not described	–	1.74% (12/688)	–	–	1.41% (28/1986)	1.22% (49/4018)
Total	10.79% (74/686)	12.21% (84/688)	15.63% (10/64)	9.20% (69/750)	9.42% (187/1986)	18.27% (734/4018)

Abbreviations: RDN, Reduning injection; TRQ, Tanreqing injection; XXN, Xixinnao injection; XYP, Xiyanping injection; YHN, Yanhuning injection.

## 4 DISCUSSION

This is the first network meta‐analysis based on Bayesian hierarchical model to assess the efficacy of the commonly used CHIs combined with azithromycin against mycoplasma pneumonia in children. A total of 167 RCTs involving 5 CHIs (Reduning injection, Tanreqing injection, Xixinnao injection, Xiyanping injection and Yanhuning injection) were included. And 8 interested outcomes were identified in this network meta‐analysis, including clinical effective rate, disappearance time of fever, cough, pulmonary rale and pulmonary shadows in X‐ray, average hospitalization time, serum level of TNF‐α and IL‐6. The results indicated that all CHIs combined with azithromycin had a superior effect than azithromycin alone among overall outcomes. Yanhuning injection combined with azithromycin ranked highest in four different outcomes and second in two based on SUCRA. Meanwhile, the results of MD and 95% CIs of concerned outcomes indicated that only Yanhuning injection combined with azithromycin had better response than other CHIs combined with azithromycin. Therefore, the efficacy of Yanhuning injection combined with azithromycin was worth paying attention to for mycoplasma pneumonia in children. In addition, cluster analysis results revealed Reduning injection combined with azithromycin was associated with a positive effect on the three group outcomes. Similarly, it was found to be the top three ranking in all outcomes based on SUCRA. Thus, Reduning injection combined with azithromycin was the other meaningful treatment. However, clinicians should choose different therapies according to the specific requirements of the patients.

As for safety, 43.11% of the RCTs did not report the monitoring of ADRs and more than half of the RCTs had ADRs occur, it suggested that there were more ADRs and less monitoring when mycoplasma pneumonia in children were treated by the included interventions. The incidence of ADRs in azithromycin alone was higher than any other intervention, which could reveal on the side face that adding CHIs to azithromycin could not increase the ADRs of the patients. The inhibitory effect of antibiotics on gastric motility leads to an increased incidence of adverse gastrointestinal reactions,^34^ which was consistent with the result that gastrointestinal reaction was in the majority from Table 3. Additionally, it is noteworthy that liver dysfunction appeared in patients who were given azithromycin, Reduning injection combined with azithromycin, Xiyanping injection combined with azithromycin and Tanreqing injection combined with azithromycin. Although the occurrence rate of Reduning injection combined with azithromycin was higher than others, no studies have shown Reduning injection can cause liver dysfunction. On the other hand, azithromycin was metabolized by liver and kidney following intravenous injection and oral medication,^35^ so long‐term medication can lead to abnormal liver function. Hence, in the treatment of mycoplasma pneumonia in children with azithromycin, whether or not combined with injection, we should not only pay attention to gastrointestinal reactions, but also pay special attention to the liver function of patients.

Azithromycin, a broad‐spectrum antibacterial and second‐generation macrolide, could inhibit bacterial protein synthesis, quorum sensing and reduces the formation of biofilm.^36^ It has longer half‐life and cellular targeting, and is used to reduce respiratory and other bacterial infections, such as gram‐positive, gram‐negative and atypical bacterial infections that lead to pneumonia.^10^, ^35^, ^37^ Azithromycin is a first‐line outpatient treatment in the case of bacterial pneumonia in the United States, or as part of a combination therapy in patients who require hospitalization.^35^, ^38^ In addition, azithromycin is often used to treat mycoplasma pneumonia in children in China. Therefore, azithromycin is a good combined therapy to evaluate the efficacy of CHIs.

There were three advantages could enhance the creditable of this study. First, this study has carried out a comprehensive retrieval. On the one hand, the CHIs included in the analysis were selected according to the eligibility criteria after a comprehensive search of all CHIs. On the other hand, this study not only retrieved electronic databases commonly used in Chinese and English, but also retrieved references from relevant literature. Second, the types of antibiotics were strictly restricted and only included azithromycin. The uniformity of intervention would reduce clinical heterogeneity to a certain extent. Third, in addition to clinical phenomena and efficacy, the hospitalization time and inflammatory indicators were also analysed. Hospitalization time can not only reflect clinical effects from side, but also reflect economic benefits. Inflammatory indicators may be related to the mechanism of drug effect. However, this study also had some limitations. First, only 20.96% (35/167) of RCTs described the method of generating random sequences. No RCT described information of allocation concealment and blinding. Therefore, the methodological quality of included RCTs was not high. Second, all RCTs were carried out in China, and the data of clinical studies in other languages were lacking. Third, there was a lack of large‐sample direct comparisons between two injections. The difference among the sample sizes of different injections would also reduce the evidence strength of the results.

## 5 WHAT IS NEW AND CONCLUSION

In summary, this study shows the comparative efficacy of CHIs combined with azithromycin for mycoplasma pneumonia in children. Among the 6 interventions, Yanhuning injection combined with azithromycin and Reduning injection combined with azithromycin were found to be more effective treatments. However, because of the limitations of this study, the results should be verified by more high‐quality large‐sample RCTs.

## CONFLICTS OF INTEREST

The authors have no conflicts of interest to declare.
