# Supplementary material for: Comparative efficacy of Chinese herbal injections combined with azithromycin for mycoplasma pneumonia in children: A Bayesian network meta‐analysis of randomized controlled trials
Source: J Clin Pharm Ther. 2019 May 22;44(5):675–84. doi: 10.1111/jcpt.12855 (PMC6852301; doi:10.1111/jcpt.12855)
Supplement: Supplementary file 2 [file JCPT-44-675-s002.doc]

**Additional file 2.** More details about the product information of 5 CHIs

| Injection name | Raw materials | Component ingredients to be measured | Botanical plant names | Labeled efficacy | Indications | Standard of authority | Adverse drug reactions |
| --- | --- | --- | --- | --- | --- | --- | --- |
| Reduning injection | *Artemisiae Annuae Herba,*  *Lonicerae Japonicae Flos,*  *Gardznize Fructus* | Gardenoside, 9.0-14.0mg/mL;  chlorogenic acid, 5.6-8.4mg/mL | *Artemisia annua* L.,  *Lonicera japonica* Thunb,  *Gardenia jasminoides* J.Ellis | Clearing heat, dispelling wind, removing toxic substance | Cold due to exogenous wind-heat, marked by high fever, headache, body pain, cough, phlegm yellow; upper respiratory tract infection and acute bronchitis | YBZ08202005 issued by China Food and Drug Administration | Dizziness, chest congestion, xerostomia, diarrhea, nausea, vomit, pruritus, skin rash, dyspnea |
| Tanreqing injection | *Scutellariae Radix*,  Bear bile powder,  Cornu gorais，  *Lonicerae Japonicae Flos,*  *Forsythiae Fructus* | Baicalin,﹥0.5mg/mL;  ursodeoxycholic Acid,﹥5.4mg/mL;  alanine, ﹥1.75-3.005.4mg/mL | *Scutellaria baicalensis* Georgi,  Selenarctos thibetanus Cuvier,  Capra hircus Linnaeus,  *Lonicera japonica* Thunb,  *Forsythia suspensa* (Thunb.) Vahl | Clearing heat-toxin, dissipating phlegm | Phlegm heat obstruct lung syndrome, such as fever, cough, expectoration, thirst, redness of tongue, and yellow fur; acute bronchitis, acute pneumonia (early) | YBZ00912003-2007Z-2009-2012 issued by China Food and Drug Administration | Dizziness, nausea, vomit, pruritus, skin rash, fever, chest congestion, edema, phlebitis, anaphylactic shock, dyspnea |
| Xixinnao injection | Asarone | Asarone, 93.0-107.0% of labelled amount | *Acorus calamus* var. *angustatus* Besser | Calm panting and suppress cough, expelling phlegm, sedation, spasmolysis, anticonvulsant | Pneumonia, bronchial asthma, chronic obstructive pulmonary disease | WS-10001-(HD-0437)-2002 issued by China Food and Drug Administration | Palpitation, chest congestion, skin rash, dizziness, nausea, vomit, anaphylactic shock |
| Xiyanping injection | Andrographolide sulfonate | Andrographolide sulfonate, 90.0%-110.0% of labelled amount | *Andrographis paniculata* (Burm.f.) Nees | Clearing heat-toxin, suppress cough and check dysentery | Bronchitis, tonsillitis, bacillary dysentery | WS-10863 (ZD-0863) -2002-2011Z issued by China Food and Drug Administration | Pruritus, skin rash, palpitation, diarrhea, vomit, diarrhea |
| Yanhuning injection | Potassium Sodium Dehydroandroandrographolide Succinate | Potassium Sodium Dehydroandroandrographolide Succinate, 90.0%-110.0% of labelled amount | *Andrographis paniculata* (Burm.f.) Nees | Clearing heat-toxin, antiviral | Viral pneumonia and viral upper respiratory tract infection | YBH06272009 issued by China Food and Drug Administration | Pruritus, skin rash, gastrointestinal adverse reactions (nausea, vomit, diarrhea), reduction of leukocyte, shiver, fever, dizziness, chest congestion, palpitation. |
