# Supplementary material for: Comparative efficacy of Chinese herbal injections combined with azithromycin for mycoplasma pneumonia in children: A Bayesian network meta‐analysis of randomized controlled trials
Source: J Clin Pharm Ther. 2019 May 22;44(5):675–84. doi: 10.1111/jcpt.12855 (PMC6852301; doi:10.1111/jcpt.12855)
Supplement: Supplementary file 3 [file JCPT-44-675-s003.doc]

**Additional file 3.** Search strategy of English databases

**PubMed**

| **#1** | Search Pneumonia, Mycoplasma[MeSH Terms] | 3690 |
| --- | --- | --- |
| **#2** | Search Primary Atypical Pneumonia[Title/Abstract] | 317 |
| **#3** | Search Mycoplasma Pneumonia[Title/Abstract] | 594 |
| **#4** | Search Mycoplasma ovipneumoniae Infection[Title/Abstract] | 6 |
| **#5** | Search Mycoplasma pneumoniae Infection[Title/Abstract] | 1055 |
| **#6** | Search Mycoplasma dispar Infection[Title/Abstract] | 1 |
| **#7** | #1 OR #2 OR #3 OR #4 OR #5 OR #6 | 4414 |
| **#8** | Search Reduning[Title/Abstract] | 40 |
| **#9** | Search Reduning injection[Title/Abstract] | 37 |
| **#10** | Search Xiyanping[Title/Abstract] | 22 |
| **#11** | Search Xiyanping injection[Title/Abstract] | 17 |
| **#12** | Search Xixinnao[Title/Abstract] | 0 |
| **#13** | Search Yanhuning[Title/Abstract] | 4 |
| **#14** | Search Yanhuning injection[Title/Abstract] | 2 |
| **#15** | Search Tanreqing[Title/Abstract] | 31 |
| **#16** | Search Tanreqing injection[Title/Abstract] | 27 |
| **#17** | Search Asarone injection[Title/Abstract] | 21 |
| **#18** | #8 OR #9 OR #10 OR #11 OR #12 OR #13 OR #14 OR #15 OR #16 OR #17 | 114 |
| **#19** | Search randomized controlled trial[Publication Type] | 460117 |
| **#20** | Search controlled clinical trial[Publication Type] | 547654 |
| **#21** | #19 OR #20 | 547654 |
| **#22** | #7 OR #18 OR #21 | 0 |

**Embase**

| #1 | Primary Atypical Pneumonia:ti,ab,kw or Mycoplasma Pneumonia:ti,ab,kw or Mycoplasma ovipneumoniae Infection:ti,ab,kw or Mycoplasma pneumoniae Infection:ti,ab,kw or Mycoplasma dispar Infection:ti,ab,kw (Word variations have been searched) | **205** |
| --- | --- | --- |
| **#2** | Reduning:ti,ab,kw or Reduning injection:ti,ab,kw or Xiyanping:ti,ab,kw or Xiyanping injection:ti,ab,kw or Xixinnao:ti,ab,kw (Word variations have been searched) | 13 |
| **#3** | Asarone injection:ti,ab,kw or Yanhuning:ti,ab,kw or Yanhuning injection:ti,ab,kw or Tanreqing:ti,ab,kw or Tanreqing injection:ti,ab,kw (Word variations have been searched) | 37 |
| **#4** | #2 or #3 | 49 |
| **#5** | randomized controlled trial:pt or Controlled clinical trial:pt (Word variations have been searched) | 534499 |
| **#6** | #1 and #4 and #5 | 0 |

**The Cochrane Library**

| **#1** | **'**primary atypical pneumonia':ab,ti OR 'mycoplasma pneumonia':ab,ti OR 'mycoplasma ovipneumoniae infection':ab,ti OR 'mycoplasma pneumoniae infection':ab,ti OR 'mycoplasma dispar infection':ab,ti | 2353 |
| --- | --- | --- |
| **#2** | 'reduning':ab,ti OR 'reduning injection':ab,ti OR 'xiyanping':ab,ti OR 'xiyanping injection':ab,ti OR 'xixinnao':ab,ti OR 'asarone injection':ab,ti OR 'yanhuning':ab,ti OR 'yanhuning injection':ab,ti OR 'tanreqing':ab,ti OR 'tanreqing injection':ab,ti | 161 |
| **#3** | #1 AND #2 | 0 |
