# Supplementary material for: Comparative efficacy of Chinese herbal injections combined with azithromycin for mycoplasma pneumonia in children: A Bayesian network meta‐analysis of randomized controlled trials
Source: J Clin Pharm Ther. 2019 May 22;44(5):675–84. doi: 10.1111/jcpt.12855 (PMC6852301; doi:10.1111/jcpt.12855)
Supplement: Supplementary file 4 [file JCPT-44-675-s004.doc]

**Additional file 4.** Details of included studies

| Study ID | Number (C/A) | Sex (M/F) | Age | Intervention | Dosage of injections | Basic treatment | Course (d) | Baseline characteristics | Outcomes |
| --- | --- | --- | --- | --- | --- | --- | --- | --- | --- |
| Zhao (2018) | 34/34 | 32/36 | C:0.5-12(6.42±1.45); A:0.5-13(6.33±1.60) | RDN+Azithromycin vs. Azithromycin | 0.5ml/(kg·d) | Abatement of fever, reducing phlegm, relieving a cough and preventing asthma | 7 | Identical | ① |
| Huang (2017) | 40/40 | 45/35 | C:2-8(4.0±1.2); A:1-8(3.9±0.7) | RDN+Azithromycin vs. Azithromycin | 1.0ml/(kg·d) | Oxygen inhalation, fluid replacement, abatement of fever, reducing phlegm and relieving a cough | 14 | Identical | ②③④⑥⑦⑧ |
| Hou (2017) | 46/46 | 55/37 | C:1-11(5.8±1.5); A:1.5-11(5.9 ±1.6) | RDN+Azithromycin vs. Azithromycin | 0.5-0.8ml/(kg·d) | Abatement of fever, reducing phlegm, relieving a cough and preventing asthma | 11 | Identical | ①②③④ |
| Shen (2017) | 41/41 | 45/37 | C:2-12(6.04 ±1.52); A:2-13(5.72±1.38) | RDN+Azithromycin vs. Azithromycin | 10-15ml/d | Abatement of fever, reducing phlegm and relieving a cough | 10 | Identical | ①②③④ |
| Tao (2017) | 45/45 | 48/42 | C:0.9-11.5(6.4±3.3); A:0.8-11.6(6.5±3.1) | RDN+Azithromycin vs. Azithromycin | 0.3-0.5ml/(kg·d) | Unspecified | 5-7 | Identical | ① |
| Fan (2016) | 34/34 | 35/33 | C:0.5-7(2.83±0.68); A:0.5-7(2.66±0.53) | RDN+Azithromycin vs. Azithromycin | 10ml/d | Abatement of fever and relieving a cough | 10 | Identical | ①②③④⑤ |
| Wang (2016) | 26/26 | 31/21 | 0.7-7(4.3±1.1) | RDN+Azithromycin vs. Azithromycin | 0.5ml/(kg·d) | NA | 13 | Identical | ①②③④⑤⑥ |
| Yang (2015) | 42/42 | 52/32 | C:1-10(5.5±2.4); A:1-10(5.8 ±2.5) | RDN+Azithromycin vs. Azithromycin | 0.5-0.8ml/(kg·d) | Abatement of fever, reducing phlegm, relieving a cough and preventing asthma | 7 | Identical | ①②③④⑥⑧ |
| Huang (2015) | 47/47 | 55/39 | C:0.5-8(5.4); A:0.6-9(4.8) | RDN+Azithromycin vs. Azithromycin | 10ml/d | NA | 14 | Identical | ①②③④⑤ |
| Zhou (2015) | 30/30 | 33/27 | C:1.2-12(5.89±2.47); A:1-14(6.26±2.59) | RDN+Azithromycin vs. Azithromycin | 0.6ml/(kg·d) | Abatement of fever, relieving a cough and preventing asthma | 12 | Identical | ①②③④ |
| Wang (2014) | 40/40 | 43/37 | 2-11 | RDN+Azithromycin vs. Azithromycin | 0.5ml/(kg·d) | Abatement of fever, reducing phlegm, relieving a cough and preventing asthma | 14 | Identical | ①②③④⑤⑥ |
| Li (2014) | 70/70 | 73/67 | C:4-13; A:4-14 | RDN+Azithromycin vs. Azithromycin | 10ml/d | Abatement of fever, reducing phlegm, relieving a cough and preventing asthma | 10 | Identical | ①②③⑤ |
| Xu (2014) | 78/74 | 81/71 | C:1-15(7.86±1.78); A:1-13(7.65±1.53） | RDN+Azithromycin vs. Azithromycin | 7.5-15ml/d | Abatement of fever, reducing phlegm, relieving a cough and preventing asthma | 14 | Identical | ① |
| Wang (2014) | 40/40 | 47/33 | C:10±1.1; A:7±1.2 | RDN+Azithromycin vs. Azithromycin | 0.5-0.8ml/(kg·d) | NA | 10 | Identical | ①②③④ |
| Wu (2013) | 49/49 | 53/45 | C:2-12(4.52±1.58); A:2-12(4. 46±1.68) | RDN+Azithromycin vs. Azithromycin | 0.5-0.7ml/(kg·d) | NA | 10 | Identical | ①②③④⑤⑥ |
| Zhang (2013) | 39/38 | 40/37 | C:0.4-3.3; A:0.3-3.2 | RDN+Azithromycin vs. Azithromycin | 0.5-1.0ml/(kg·d) | Unspecified | 7 | Identical | ①②③④ |
| Wei (2013) | 30/30 | 37/23 | 4-15 | RDN+Azithromycin vs. Azithromycin | 10-15ml/d | NA | 14 | Unspecified | ① |
| Huo (2013) | 40/40 | 43/37 | C:1-12(5.8±1.9); A:1-12(5.7±2.3) | RDN+Azithromycin vs. Azithromycin | 0.5ml/(kg·d) | Abatement of fever, reducing phlegm and relieving a cough | 21 | Identical | ①②③⑤ |
| Dai (2012) | 41/41 | 44/38 | C:1-11(6.4±2.5); A:1-14(6.8 ±2.6) | RDN+Azithromycin vs. Azithromycin | 0.6-0.8ml/(kg·d) | Abatement of fever, reducing phlegm, relieving a cough and preventing asthma | 14 | Identical | ①②③④⑧ |
| An (2012) | 80/84 | 88/76 | C:1-8; A:1-8 | RDN+Azithromycin vs. Azithromycin | 10ml/d | NA | 14 | Identical | ①②③④⑤ |
| Xu (2011) | 20/20 | 24/16 | C:3-13; A:3-12 | RDN+Azithromycin vs. Azithromycin | 0.5-0.8ml/(kg·d) | NA | 14 | Identical | ① |
| Du (2011) | 48/48 | 59/37 | 0.4-15 | RDN+Azithromycin vs. Azithromycin | 0.5ml/(kg·d) | NA | 11 | Identical | ①②③④ |
| Liu (2011) | 88/88 | 101/75 | 3.5-10 | RDN+Azithromycin vs. Azithromycin | 10ml/d | NA | 14 | Identical | ①②③④ |
| Duan (2011) | 30/30 | 35/25 | 3-13 | RDN+Azithromycin vs. Azithromycin | 10-15ml/d | NA | 14 | Unspecified | ① |
| Song (2011) | 45/40 | 47/38 | C:3-12; A:2-13 | RDN+Azithromycin vs. Azithromycin | 0.5ml/(kg·d) | NA | 7 | Unspecified | ①②③④⑤ |
| Shi (2009) | 40/40 | 45/35 | C:3-12; A:3-12 | RDN+Azithromycin vs. Azithromycin | 0.5-1.0ml/(kg·d) | NA | 14 | Identical | ①②③④⑥ |
| Lu (2008) | 58/56 | 61/53 | C:1-12; A:1-12 | RDN+Azithromycin vs. Azithromycin | 0.5ml/(kg·d) | NA | 14 | Identical | ①②③⑤ |
| Zhu (2018) | 60/60 | 62/58 | C:1-12(6.93±1.48); A:1-13(6.87 ±1.54) | RDN+Azithromycin vs. Azithromycin | 0.5ml/(kg·d) | Reducing phlegm, relieving a cough and preventing asthma | 21 | Identical | ②③④⑦ |
| Zhang (2017) | 40/40 | 44/36 | C:5-14(8.4± 2.2); A:5-13(8.1± 1.9) | RDN+Azithromycin vs. Azithromycin | 10ml/d | Abatement of fever, reducing phlegm and preventing asthma | 7 | Identical | ① |
| Wang (2016) | 40/40 | 44/36 | C:0.6-12(6.54±3.21); A:0.6-11.0( 6.37±2.85) | RDN+Azithromycin vs. Azithromycin | 0.3-0.5ml/(kg·d) | Abatement of fever, reducing phlegm, relieving a cough and preventing asthma | 6-8 | Identical | ①②③④⑤⑦⑧ |
| Gao (2015) | 18/18 | 19/17 | 3-11(5.6±1.8) | RDN+Azithromycin vs. Azithromycin | 10ml/d | NA | 7 | Identical | ①②③④ |
| Chen (2015) | 41/41 | 47/35 | C:1-12(6.6±1.1); A:2-11(6.8±1.4) | RDN+Azithromycin vs. Azithromycin | 0.5ml/(kg·d) | Abatement of fever, reducing phlegm, relieving a cough and preventing asthma | 14 | Identical | ①②③④⑤ |
| Chen (2014) | 60/60 | 65/55 | C:2-11; A:2-11 | RDN+Azithromycin vs. Azithromycin | 1.0ml/(kg·d) | Abatement of fever, reducing phlegm and relieving a cough | 14 | Identical | ①⑦ |
| Yong (2017) | 29/29 | 30/28 | C:1-3(1.7±0.6); A:1-3(1.8±0.4) | XYP+Azithromycin vs. Azithromycin | 2mg/(kg·d) | Reducing phlegm and preventing asthma | 7 | Identical | ①⑦⑧ |
| Li (2017) | 60/60 | 75/45 | C:1-11(5.7±0.7); A:2-9(5.3±2.7) | XYP+Azithromycin vs. Azithromycin | 5-10mg/(kg·d) or 0.2-0.4ml/(kg·d) | Oxygen inhalation, regulating water electrolyte, relieving a cough and preventing asthma | 8 | Identical | ②③④⑤ |
| Dou (2016) | 40/40 | 44/36 | C:2-10(5.3±2.4); A:3-9.8(4.9±1.9) | XYP+Azithromycin vs. Azithromycin | 2mg/(kg·d) | Abatement of fever, reducing phlegm and preventing asthma | 14 | Identical | ①⑦⑧ |
| Wang (2016) | 40/39 | 46/33 | C:2-14(9±1.1) ; A: 2.1-14(9±0.9） | XYP+Azithromycin vs. Azithromycin | 100mg/d | Abatement of fever, reducing phlegm, relieving a cough and preventing asthma | 10 | Identical | ①②③④ |
| Guan (2016) | 50/50 | 69/31 | C:(4.7±1.3); A:4.6±1.4 | XYP+Azithromycin vs. Azithromycin | 10mg/(kg·d) | Abatement of fever, relieving a cough and preventing asthma | 15 | Identical | ① |
| Yu (2015) | 50/50 | 47/53 | C:2-12(7.2±2.5); A:2-13(6.3±2.8) | XYP+Azithromycin vs. Azithromycin | 10mg/(kg·d) | Unspecified | 7 | Identical | ①⑦⑧ |
| Li (2015) | 40/40 | 44/36 | C:1.8-7.7(4.2±1.5); A:1.2-7.5(4.4±1.3) | XYP+Azithromycin vs. Azithromycin | 0.4ml/(kg·d) | NA | 7 | Identical | ①②③④⑤ |
| Lin (2015) | 30/30 | 23/37 | C:1-12(6.4±1.3); A:1-12(6.3±1.4) | XYP+Azithromycin vs. Azithromycin | 2mg/(kg·d) | Abatement of fever, relieving a cough and preventing asthma | 7 | Identical | ①②③④ |
| Zhang (2015) | 44/43 | 42/45 | 1-14(6.63±2.05) | XYP+Azithromycin vs. Azithromycin | 8mg/(kg·d) | Abatement of fever, reducing phlegm and relieving a cough | 7 | Identical | ②③④⑤ |
| Zuo (2015) | 51/51 | 58/44 | 0.5-13(7.29±1.46) | XYP+Azithromycin vs. Azithromycin | 0.2-0.4ml/(kg·d) | Unspecified | 5 | Identical | ① |
| Li (2015) | 51/51 | 55/47 | C:0.75-12(7.2); A:0.6-13(7.6) | XYP+Azithromycin vs. Azithromycin | 0.2-0.4ml/(kg·d) | Oxygen inhalation, fluid replacement, sedation | 7 | Identical | ① |
| Liang (2015) | 49/49 | 58/40 | C:0.5-11(4.9±1.8); A:0.6-10(4.3±1.6) | XYP+Azithromycin vs. Azithromycin | 10mg/(kg·d) | Abatement of fever, reducing phlegm, relieving a cough and preventing asthma, nutritional support | 8 | Identical | ①②③④⑤⑥⑦⑧ |
| Yang (2014) | 63/63 | 73/53 | C:2-13(5±3); A:2-13(5±3) | XYP+Azithromycin vs. Azithromycin | 0.2-0.4ml/(kg·d) | Abatement of fever, reducing phlegm, relieving a cough and preventing asthma | 14 | Unspecified | ①②③④⑤ |
| Ning (2014) | 60/60 | 74/46 | C:(5.2±1.3); A:5.3±1.4 | XYP+Azithromycin vs. Azithromycin | 2mg/(kg·d) | Oxygen inhalation, abatement of fever, reducing phlegm, relieving a cough and preventing asthma | 7 | Identical | ①②③④⑤ |
| Jisng (2013) | 44/40 | 45/39 | C:0.6-12(6.3±1.6); A:1-13(5.6±1.3) | XYP+Azithromycin vs. Azithromycin | 2mg/(kg·d) | Reducing phlegm, relieving a cough and preventing asthma, spasmolysis | 7 | Identical | ①②③④⑤ |
| Chen (2013) | 28/28 | - | 2-12(7.5) | XYP+Azithromycin vs. Azithromycin | 0.2-0.4ml/(kg·d) | Unspecified | 11 | Identical | ① |
| Wang (2013) | 60/60 | 69/51 | 0.5-12(8.1±1.6) | XYP+Azithromycin vs. Azithromycin | 10mg/(kg·d) | Abatement of fever, reducing phlegm, relieving a cough and preventing asthma, nutritional support | 8 | Identical | ②③④⑤⑦⑧ |
| Sun (2012) | 60/60 | 62/58 | 0.5-6(4.8±1.4) | XYP+Azithromycin vs. Azithromycin | 0.2-0.4ml/(kg·d) | Unspecified | 8 | Identical | ①②③⑥ |
| Du (2011) | 47/43 | 48/42 | C:3.6±1.7; A:3.8±1.4 | XYP+Azithromycin vs. Azithromycin | 10mg/(kg·d) | Abatement of fever, reducing phlegm, relieving a cough and preventing asthma | 7 | Identical | ① |
| Deng (2015) | 56/54 | 67/43 | C:5.49±1.64; A:5.98±1.50 | XYP+Azithromycin vs. Azithromycin | 1-3mg/(kg·d) | Unspecified | 14 | Identical | ①②③④⑤⑦⑧ |
| Zhang (2014) | 30/30 | 45/15 | 1-10 | XXN+Azithromycin vs. Azithromycin | 0.5mg/(kg·d) | NA | 14 | Identical | ① |
| Yao (2011) | 25/25 | 26/24 | C:4.7±1.3; A:5.1±1.9 | XXN+Azithromycin vs. Azithromycin | 0.5mg/(kg·d) | Abatement of fever, relieving a cough, regulating water electrolyte | 14 | Identical | ①②③④ |
| Yin (2009) | 35/32 | 34/33 | C:5.08±2.58; A:5.14±2.39 | XXN+Azithromycin vs. Azithromycin | 0.5mg/(kg·d) | NA | 14 | Identical | ①②③④⑤ |
| Song (2005) | 50/48 | 53/45 | C:1-13; A:1-13 | XXN+Azithromycin vs. Azithromycin | 0.5-1.0mg/(kg·d) | NA | 10 | Identical | ①③④ |
| Wang (2018) | 51/51 | 52/50 | C:6.52±2.48; A:6.49±2.35 | YHN+Azithromycin vs. Azithromycin | 5mg/(kg·d) | Abatement of fever, relieving a cough | 10 | Identical | ① |
| Guo (2017) | 65/65 | 69/61 | C:6.7±0.8; A:6.6±0.5 | YHN+Azithromycin vs. Azithromycin | 5mg/(kg·d) | Reducing phlegm, relieving a cough | 7 | Identical | ⑦⑧ |
| Li (2017) | 23/23 | 27/19 | 3-15(5.2±1.6) | YHN+Azithromycin vs. Azithromycin | 6mg/(kg·d) | relieving a cough and preventing asthma | 7 | Identical | ①②③④ |
| Liu (2017) | 30/30 | 31/29 | C:0.4-12(7.1±2.5); A:0.5-11(7.3±2.8) | YHN+Azithromycin vs. Azithromycin | 5-10mg/(kg·d) | Unspecified | 14 | Identical | ①②③④⑥ |
| Yu (2017) | 40/40 | 48/32 | C:0.5-12(5.5±1.5); A:0.5-12(5.9±1.2) | YHN+Azithromycin vs. Azithromycin | 8-12mg/(kg·d) | NA | 14 | Identical | ① |
| Lan (2016) | 81/81 | 83/79 | C:1-12(5.9±3.4); A:1-13(6.1±3.6) | YHN+Azithromycin vs. Azithromycin | 5-10mg/(kg·d) | NA | 14 | Identical | ① |
| Yang (2016) | 50/50 | 62/38 | C:0.8-14(6.2±2.1); A:0.9-13(6.7±2.2) | YHN+Azithromycin vs. Azithromycin | 5mg/(kg·d) | Reducing phlegm, relieving a cough | 7 | Identical | ① |
| Han (2015) | 27/26 | 31/22 | C:1-8(5.6±1.8); A:2-9(6.1±1.5) | YHN+Azithromycin vs. Azithromycin | 10mg/(kg·d) | Abatement of fever, reducing phlegm and preventing asthma | 15 | Identical | ②③④⑤ |
| Li (2015) | 40/40 | 47/33 | C:0.5-8(2.54±1.46); A:0.6-7(2.49±1.37) | YHN+Azithromycin vs. Azithromycin | 10mg/(kg·d) | NA | 7 | Identical | ①②③④ |
| Ma (2015) | 62/64 | 94/32 | C:7±1.6; A:7±1.3 | YHN+Azithromycin vs. Azithromycin | 10mg/(kg·d) | NA | 16 | Identical | ①④ |
| Ye (2015) | 40/40 | 49/31 | C:0.8-13(5.81±1.30); A:1-14(5.86±1.32) | YHN+Azithromycin vs. Azithromycin | 10mg/(kg·d) | Unspecified | 22 | Identical | ① |
| Zhong (2015) | 55/55 | 63/47 | C:2.4-14.2(5.4±1.2); A:1.6-11.4(4.9±2.6) | YHN+Azithromycin vs. Azithromycin | 10mg/(kg·d) | NA | 12 | Identical | ⑦⑧ |
| Dong (2014) | 40/40 | 42/38 | 0.25-7 | YHN+Azithromycin vs. Azithromycin | ≤10mg/(kg·d) | NA | 14 | Identical | ① |
| Wen (2014) | 40/39 | 56/23 | C:6.0±1.0; A:6.2±1.1 | YHN+Azithromycin vs. Azithromycin | 10mg/(kg·d) | Abatement of fever, relieving a cough and preventing asthma | 10 | Identical | ②③④⑤⑥⑦⑧ |
| Zhang (2014) | 32/32 | 33/31 | C:2-14(5.6±1.7); A:2-14(5.5±1.8) | YHN+Azithromycin vs. Azithromycin | 10mg/(kg·d) | Abatement of fever, reducing phlegm, relieving a cough | 7 | Identical | ①⑦⑧ |
| Zhang (2014) | 52/52 | 61/43 | 5-11(5.3±1.2) | YHN+Azithromycin vs. Azithromycin | 5-10mg/(kg·d) | Reducing phlegm, relieving a cough, regulating water electrolyte | 12 | Identical | ①②③④⑥ |
| Zhang (2014) | 43/42 | 56/29 | C:0.5-11(7.2±2.1); A:0.5-12(7.5±2.6) | YHN+Azithromycin vs. Azithromycin | 5-10mg/(kg·d) | Unspecified | 14 | Identical | ①②③④⑥ |
| Zhang (2012) | 24/24 | 28/20 | C:3-12; A:3-12 | YHN+Azithromycin vs. Azithromycin | 10mg/(kg·d) | Abatement of fever, reducing phlegm, relieving a cough and preventing asthma | 7 | Identical | ① |
| Zhou (2012) | 47/47 | 65/29 | C:4.7士1.2; A:5.0±1.4 | YHN+Azithromycin vs. Azithromycin | 5-10mg/(kg·d) | NA | 14 | Identical | ①②③④⑥ |
| Wang (2012) | 167/156 | 176/147 | C:5.1±2.0; A:5.3±1.8 | YHN+Azithromycin vs. Azithromycin | 10mg/(kg·d) | NA | 7 | Identical | ①④⑤⑥ |
| Yan (2012) | 40/40 | 44/36 | C:4.5; A:5.5 | YHN+Azithromycin vs. Azithromycin | 10mg/(kg·d) | NA | 7 | Identical | ① |
| Cao (2011) | 36/36 | 41/31 | C:0.5-12(6.3±2.5); A:0.4-12(6.1±2.8) | YHN+Azithromycin vs. Azithromycin | 5-10mg/(kg·d) | Reducing phlegm, relieving a cough and preventing asthma | 5-7 | Identical | ①②③④⑥ |
| Feng (2011) | 53/50 | 54/49 | C:2-12; A:2-12 | YHN+Azithromycin vs. Azithromycin | 5-10mg/(kg·d) | NA | 14 | Identical | ① |
| Liu (2011) | 48/48 | 57/39 | 2-14(6.5±1.0) | YHN+Azithromycin vs. Azithromycin | 0.24g/(kg·d) | NA | 10 | Identical | ①②③④ |
| Song (2011) | 79/73 | 83/69 | C:3-15(5.2±1.3); A:3-15(5.2±1.3) | YHN+Azithromycin vs. Azithromycin | 8-12mg/(kg·d) | NA | 7 | Identical | ①②③④⑥ |
| Su (2011) | 60/56 | 64/52 | C:0.8-14(5.86±1.32); A:0.8-14(5.81±1.30) | YHN+Azithromycin vs. Azithromycin | 8-10mg/(kg·d) | NA | 7 | Identical | ①②③④⑥ |
| Lv (2011) | 45/40 | 47/38 | C:3-12; A:2-13 | YHN+Azithromycin vs. Azithromycin | 5-10mg/(kg·d) | NA | 18 | Identical | ①②③④⑤ |
| Cui (2010) | 58/58 | 69/47 | C:1-14; A:1-14 | YHN+Azithromycin vs. Azithromycin | 8-12mg/(kg·d) | NA | 15 | Identical | ①③④⑤⑥ |
| Li (2010) | 64/50 | 67/47 | C:4.6; A:4.6 | YHN+Azithromycin vs. Azithromycin | 5-10mg/(kg·d) | NA | 14 | Identical | ① |
| Luo (2010) | 60/60 | 74/46 | C:3-12; A:3-12 | YHN+Azithromycin vs. Azithromycin | 5-10mg/(kg·d) | NA | 14 | Identical | ① |
| Meng (2010) | 42/33 | 39/36 | C:1-13(7.5±4.8); A:1-13(7.4±4.9) | YHN+Azithromycin vs. Azithromycin | 5mg/(kg·d) | NA | 14 | Identical | ①②⑤ |
| Jiang (2010) | 46/42 | 48/40 | C:1-14; A:1-14 | YHN+Azithromycin vs. Azithromycin | 10mg/(kg·d) | NA | 5-7 | Identical | ①③④ |
| Cao (2009) | 45/41 | 47/39 | 0.6-14(4±2) | YHN+Azithromycin vs. Azithromycin | 10mg/(kg·d) | NA | 7 | Identical | ②③④ |
| Jiang (2008) | 68/64 | 72/60 | C:1-14; A:1-14 | YHN+Azithromycin vs. Azithromycin | 5-10mg/(kg·d) | NA | 7 | Identical | ①②③⑤ |
| Chen (2007) | 71/70 | 76/65 | C:0.8-12; A:0.8-12 | YHN+Azithromycin vs. Azithromycin | 5-10mg/(kg·d) | NA | 5-7 | Identical | ①②③⑤ |
| Zhou (2017) | 75/75 | 74/76 | C:6.4-5.5(2.5±1.2); A:0.4-6.4(3.0±1.5) | TRQ+Azithromycin vs. Azithromycin | 0.3-0.5mg/(kg·d) | NA | 14 | Identical | ① |
| Cheng (2017) | 40/40 | 47/33 | C:1-5; A:1.16-5 | TRQ+Azithromycin vs. Azithromycin | 0.5mg/(kg·d) | NA | 14 | Identical | ⑦ |
| Liu (2017) | 47/47 | 56/38 | C:0.92-8(4.82±1.09); A:0.8-8(4.69±1.12) | TRQ+Azithromycin vs. Azithromycin | 0.3-0.5mg/(kg·d) | NA | 11 | Identical | ①②④⑤⑦ |
| Lin (2017) | 41/41 | 38/44 | C:0.16-3(1.5±0.3); A:0.16-3(1.5±0.3) | TRQ+Azithromycin vs. Azithromycin | 0.5-1.0mg/(kg·d) | NA | 14 | Identical | ①②③④⑤ |
| Zhang (2017) | 65/65 | 69/61 | C:0.3-11(5.46±2.75); A:0.4-10(5.36±2.78) | TRQ+Azithromycin vs. Azithromycin | 0.3-0.5mg/(kg·d) | Reducing phlegm and preventing asthma, antiallergic | 7 | Identical | ①②③④⑤ |
| Li (2017) | 39/39 | 48/30 | C:3-12(6.5±1.2); A:2-13(6.3±1.7) | TRQ+Azithromycin vs. Azithromycin | 0.3-0.5mg/(kg·d) | NA | 14 | Identical | ① |
| Lin (2017) | 36/36 | 40/32 | C:2.7-10.5(6.9±2.1); A:3.1-11.2(7.1±2.5) | TRQ+Azithromycin vs. Azithromycin | 0.3-0.5mg/(kg·d) | Unspecified | 21 | Identical | ①②③④⑦⑧ |
| Wu (2017) | 80/80 | 89/71 | C:1-12(6.75±3.26); A:1-12(5.75±4.13 ) | TRQ+Azithromycin vs. Azithromycin | 0.3-0.5mg/(kg·d) | NA | 7-11 | Identical | ①②③④⑥ |
| Zhang (2016) | 39/37 | 46/30 | C:4-12(7.2±1.8); A:5-14(7.6±2.2) | TRQ+Azithromycin vs. Azithromycin | 0.5mg/(kg·d) | NA | 14 | Identical | ①②③⑤ |
| Jia (2016) | 43/42 | 47/38 | 0.4-12(2.4±0.3) | TRQ+Azithromycin vs. Azithromycin | 0.3-0.5mg/(kg·d) | Reducing phlegm, relieving a cough and preventing asthma | 14 | Identical | ①②③④ |
| Zhao (2016) | 37/37 | 35/39 | C:2-14(7.55±3.21); A:2-13(7.62±3.33) | TRQ+Azithromycin vs. Azithromycin | 0.3-0.5mg/(kg·d) | NA | 14 | Identical | ① |
| Yu (2016) | 93/93 | 103/83 | C:0.7-7(3.48±1.03); A:0.75-6.5(3.51±1.05) | TRQ+Azithromycin vs. Azithromycin | 0.3-0.5mg/(kg·d) | Abatement of fever, reducing phlegm and preventing asthma, nutritional support | 14 | Identical | ①②③⑤⑥ |
| Wang (2016) | 60/60 | 66/54 | C:0.5-12(2.4); A:0.55-11(2.6) | TRQ+Azithromycin vs. Azithromycin | 0.3-0.5mg/(kg·d) | Abatement of fever, reducing phlegm, relieving a cough | 10 | Identical | ①②③④ |
| Liu (2016) | 71/71 | 81/61 | 0.4-12(6.05±1.34) | TRQ+Azithromycin vs. Azithromycin | 0.5mg/(kg·d) | Abatement of fever, relieving a cough | 14 | Identical | ①②③④ |
| Han (2016) | 50/50 | 54/46 | C:0-15(7.3±1.0); A:0-15(7.3±1.0) | TRQ+Azithromycin vs. Azithromycin | 0.5mg/(kg·d) | NA | 15 | Identical | ① |
| Zhang (2016) | 43/43 | 54/32 | C:0.5-13; A:0.5-13 | TRQ+Azithromycin vs. Azithromycin | 0.5mg/(kg·d) | Abatement of fever, reducing phlegm, relieving a cough | 14 | Identical | ①②③④⑤ |
| Liu (2016) | 35/33 | 34/34 | 1-10(5.13±1.72) | TRQ+Azithromycin vs. Azithromycin | 0.3-0.5mg/(kg·d) | NA | 14 | Identical | ①②③④ |
| Guo (2016) | 42/42 | 45/39 | C:1-14(6±3); A:1-14(6.6±1.3) | TRQ+Azithromycin vs. Azithromycin | 0.5mg/(kg·d) | NA | 7 | Identical | ①②③④⑥ |
| Qi (2016) | 43/43 | 45/41 | 1-9(5.73±1.06) | TRQ+Azithromycin vs. Azithromycin | 0.3-0.5mg/(kg·d) | Oxygen inhalation, abatement of fever, reducing phlegm, relieving a cough | 7d | Identical | ①②③④⑤ |
| Li (2015) | 64/64 | 60/68 | C:3-12(5.0±2.0); A:2-11(4.5± 2.5) | TRQ+Azithromycin vs. Azithromycin | 0.5mg/(kg·d) | NA | 14 | Identical | ①②③④⑤ |
| Hu (2015) | 70/70 | 81/59 | C:2-14(9±1.37); A:1-13(8±1.65) | TRQ+Azithromycin vs. Azithromycin | 0.3-0.5mg/(kg·d) | Abatement of fever, relieving a cough and preventing asthma | 7 | Identical | ①②③④ |
| Deng (2015) | 30/30 | 37/23 | C:1-14(5.7±2.3); A:1-12(5.3±2.6) | TRQ+Azithromycin vs. Azithromycin | 0.3-0.5mg/(kg·d) | NA | 14 | Identical | ① |
| Yi (2015) | 32/32 | 35/29 | C:3.2-12.5(6.8±3.1); A:3.5-12.5(6.3±2.9) | TRQ+Azithromycin vs. Azithromycin | 0.3-0.5mg/(kg·d) | NA | 7 | Identical | ①②③④⑥ |
| Fang (2015) | 67/67 | 72/62 | C:0.4-14(6.15±0.26); A:0.58-13(6.08±0.33) | TRQ+Azithromycin vs. Azithromycin | 0.3-0.5mg/(kg·d) | NA | 14 | Identical | ①②③④⑥⑦⑧ |
| Li (2015) | 34/33 | 33/34 | C:0.25-6(3.2±1.5); A:0.3-6(3.3±1.4) | TRQ+Azithromycin vs. Azithromycin | 0.5mg/(kg·d) | Abatement of fever, relieving a cough and preventing asthma | 14 | Identical | ①②③④⑤ |
| Chen (2015) | 35/35 | 39/31 | C:0.9-12(5.6±2.1); A:0.8-11(5.6±2.3) | TRQ+Azithromycin vs. Azithromycin | 0.3-0.5mg/(kg·d) | Abatement of fever, reducing phlegm and preventing asthma | 14 | Identical | ① |
| Yuan (2015) | 55/55 | 60/50 | C:2-11(6.5±1.4); A:3-12(6.2±1.1) | TRQ+Azithromycin vs. Azithromycin | 0.5mg/(kg·d) | NA | 14 | Identical | ①②③④ |
| Wen (2015) | 30/30 | 29/31 | C:0.5-12(6.5±2.8); A:0.5-12(6.3±3.2) | TRQ+Azithromycin vs. Azithromycin | 10ml | NA | 14 | Identical | ① |
| Wu (2014) | 40/40 | 39/41 | C:0.4-12(7.75±4.72); A:0.5-11(7.68±4.52) | TRQ+Azithromycin vs. Azithromycin | 0.5mg/(kg·d) | NA | 15 | Identical | ①②③④ |
| Mei (2014) | 41/41 | 45/37 | 1.5-14 | TRQ+Azithromycin vs. Azithromycin | 0.5mg/(kg·d) | Abatement of fever, reducing phlegm, relieving a cough and preventing asthma | 35 | Identical | ①②③④ |
| Huang (2014) | 50/50 | 53/47 | C:0.8-13(8.13±3.41); A:0.8-13(8.14±3.56) | TRQ+Azithromycin vs. Azithromycin | 0.3-0.5mg/(kg·d) | NA | 14 | Identical | ①②③④⑥ |
| Hu (2014) | 35/35 | 38/32 | C:0.6-11.8(4.3±1.7); A:0.8-10.5(3.4±1.5) | TRQ+Azithromycin vs. Azithromycin | 0.5mg/(kg·d) | NA | 14 | Identical | ①②③⑥ |
| Fu (2013) | 64/64 | 77/51 | 1-11 | TRQ+Azithromycin vs. Azithromycin | 0.3-0.5mg/(kg·d) | NA | 14 | Identical | ①②③④ |
| Hu (2014) | 45/45 | 47/43 | C:1-14(6.4±3.1); A:1-14(6.6±3.2) | TRQ+Azithromycin vs. Azithromycin | 0.3-0.5mg/(kg·d) | NA | 11 | Identical | ①②③④⑥ |
| Lu (2014) | 44/42 | 49/37 | 1-12(4.6±3.7) | TRQ+Azithromycin vs. Azithromycin | 0.3-0.5mg/(kg·d) | NA | 14 | Identical | ①②③④ |
| Li (2014) | 40/40 | 43/37 | C:0.8-13; A:0.9-13 | TRQ+Azithromycin vs. Azithromycin | 0.3-0.5mg/(kg·d) | NA | 14 | Identical | ①②③④ |
| Zhen (2014) | 38/38 | 41/35 | C:0.5-13; A:0.45-12 | TRQ+Azithromycin vs. Azithromycin | 10ml | NA | 14 | Identical | ①②③④ |
| Xing (2014) | 50/50 | 47/53 | C:1.4-13.1(6.5±2.4); A:1.5-12.9(6.6±2.3) | TRQ+Azithromycin vs. Azithromycin | 0.5mg/(kg·d) | NA | 14 | Identical | ① |
| Sheng (2014) | 34/34 | 37/31 | 0.75-13 | TRQ+Azithromycin vs. Azithromycin | 0.5mg/(kg·d) | NA | 19 | Identical | ①②③④ |
| Yao (2014) | 74/74 | 76/72 | C:0.3-11(5.1±1.3); A:0.4-11(5.2±1.1) | TRQ+Azithromycin vs. Azithromycin | 0.5mg/(kg·d) | NA | 14 | Identical | ①②④⑤ |
| Bai (2013) | 60/60 | 63/57 | C:5.1-13(6.4); A:5-13(6.5) | TRQ+Azithromycin vs. Azithromycin | 0.3-0.5mg/(kg·d) | Abatement of fever, reducing phlegm, relieving a cough and preventing asthma | 10 | Identical | ①②③⑤ |
| Chen (2013) | 42/42 | 46/38 | 1-13(3.37±2.43) | TRQ+Azithromycin vs. Azithromycin | 0.3-0.5mg/(kg·d) | NA | 14 | Identical | ① |
| Yuan (2013) | 41/41 | 47/35 | C:3-14(6.48．2.93); A:2.5-13(6.25±3.14) | TRQ+Azithromycin vs. Azithromycin | 0.5mg/(kg·d) | NA | 14 | Identical | ①②③④ |
| Wang (2013) | 28/26 | 29/25 | C:0.8-3; A:0.8-3 | TRQ+Azithromycin vs. Azithromycin | 0.5mg/(kg·d) | Abatement of fever, reducing phlegm, relieving a cough and preventing asthma | 7 | Identical | ①②③ |
| Liu (2013) | 32/32 | 29/35 | 0.5-12 | TRQ+Azithromycin vs. Azithromycin | 0.5mg/(kg·d) | Abatement of fever, reducing phlegm, relieving a cough and preventing asthma | 14 | Identical | ①②③④⑤ |
| Wang (2013) | 40/40 | 40/40 | C:1-14(6.5±3.2); A:1-14(6.7±3.2) | TRQ+Azithromycin vs. Azithromycin | 0.3-0.5mg/(kg·d) | NA | 14 | Identical | ①②③④⑥ |
| Zhan (2013) | 46/42 | 48/40 | 0.5-14 | TRQ+Azithromycin vs. Azithromycin | 0.5mg/(kg·d) | NA | 15 | Identical | ①⑦⑧ |
| Wang (2013) | 60/60 | 61/59 | C:3.4-11.6(7.4±3.6); A:3.2-11.5(7.6±3.2) | TRQ+Azithromycin vs. Azithromycin | 0.3-0.5mg/(kg·d) | Abatement of fever, reducing phlegm, relieving a cough | 14 | Identical | ①②③④⑦⑧ |
| Wei (2013) | 60/60 | 64/56 | C:3-8(5.0±0.5); A:3-7(4.5±0.8) | TRQ+Azithromycin vs. Azithromycin | 0.3-0.5mg/(kg·d) | NA | 14 | Identical | ①②③④ |
| Ma (2013) | 62/62 | 73/51 | 2-13 | TRQ+Azithromycin vs. Azithromycin | 0.3-0.5mg/(kg·d) | NA | 14 | Identical | ①②③④⑤ |
| Cui (2013) | 38/38 | 46/30 | 2-13 | TRQ+Azithromycin vs. Azithromycin | 0.3-0.5mg/(kg·d) | Abatement of fever, reducing phlegm, relieving a cough, fluid replacement | 14 | Identical | ①②③④⑤ |
| Wang (2012) | 45/48 | 55/38 | C:1-12(3.17±1.49); A:1-13(3.77±1.56) | TRQ+Azithromycin vs. Azithromycin | 0.3-0.5mg/(kg·d) | NA | 14 | Identical | ① |
| Wang (2013) | 45/45 | 48/42 | C:5.5±4.2; A:5.6±4.0 | TRQ+Azithromycin vs. Azithromycin | 0.3-0.5mg/(kg·d) | NA | 10 | Identical | ①②③④ |
| Li (2012) | 50/50 | 51/49 | C:2-12(4.46±1.68); A:2-12(4.52±1.58) | TRQ+Azithromycin vs. Azithromycin | 0.5mg/(kg·d) | NA | 14 | Identical | ① |
| Cheng (2012) | 116/122 | - | 0-13 | TRQ+Azithromycin vs. Azithromycin | 0.3-0.5mg/(kg·d) | NA | 14 | Unspecified | ①②③④ |
| Wang (2012) | 45/45 | 55/35 | C:1-12(3.17±1.49); A:1-13(3.77±1.56) | TRQ+Azithromycin vs. Azithromycin | 0.3-0.5mg/(kg·d) | NA | 14 | Identical | ① |
| Zi (2012) | 53/53 | 58/48 | C:1-14(6.23±1.13); A:1-14(5.80±1.23) | TRQ+Azithromycin vs. Azithromycin | 5-10ml/(kg·d) | Reducing phlegm | 10 | Identical | ①②③ |
| Zhang (2012) | 40/40 | 50/30 | 1-14 | TRQ+Azithromycin vs. Azithromycin | 5ml/(kg·d) | NA | 7 | Identical | ①②③④ |
| Xiao (2011) | 50/50 | 43/57 | C:0.75-15(3.86±2.7); A:0.58-14(3.90±2.9) | TRQ+Azithromycin vs. Azithromycin | 0.5mg/(kg·d) | NA | 21 | Identical | ①②③⑥ |
| Zhang (2011) | 40/40 | 47/33 | 0.8-12(4.97±1.23) | TRQ+Azithromycin vs. Azithromycin | 0.5mg/(kg·d) | NA | 21 | Identical | ①②③④⑥ |
| Liao (2011) | 42/40 | 48/34 | C:3-12; A:3-12 | TRQ+Azithromycin vs. Azithromycin | 0.5-1.0mg/(kg·d) | NA | 14 | Identical | ① |
| Cao (2011) | 124/124 | 146/102 | 3-12(4.67) | TRQ+Azithromycin vs. Azithromycin | 0.5-1.0mg/(kg·d) | NA | 14 | Identical | ①②③④⑤ |
| Xu (2011) | 60/60 | 59/61 | C:7.37±3.61); A:8.01±2.97 | TRQ+Azithromycin vs. Azithromycin | 0.3-0.5mg/(kg·d) | NA | 21 | Identical | ①②③④⑥ |
| Zhang (2011) | 32/32 | 40/24 | 1-14 | TRQ+Azithromycin vs. Azithromycin | 5-15ml | NA | 5-7 | Identical | ①②③④ |
| Wang (2011) | 45/35 | 45/35 | 4-14 | TRQ+Azithromycin vs. Azithromycin | 0.3-0.5mg/(kg·d) | NA | 7 | Identical | ① |
| Fan (2011) | 75/75 | 81/69 | 0.5-13(5.6) | TRQ+Azithromycin vs. Azithromycin | 20ml | Abatement of fever, reducing phlegm, relieving a cough | 21 | Identical | ①②③④ |
| Du (2011) | 30/30 | 29/31 | C:0.3-2.5; A:0.41-2.66 | TRQ+Azithromycin vs. Azithromycin | 0.3-0.5mg/(kg·d) | NA | 16 | Identical | ①②③④ |
| Yan (2010) | 68/56 | 65/59 | C:3.25±1.24; A:3.47±1.38 | TRQ+Azithromycin vs. Azithromycin | 0.3-0.5mg/(kg·d) | NA | 14 | Identical | ①②③④ |
| He (2009) | 28/28 | 31/25 | C:1-14(3.43±1.53); A:1-15(3.67±1.49) | TRQ+Azithromycin vs. Azithromycin | 10ml | NA | 21 | Identical | ① |
| Shang (2009) | 40/40 | 45/35 | C:3-12; A:3-12 | TRQ+Azithromycin vs. Azithromycin | 0.5-1.0mg/(kg·d) | NA | 14 | Identical | ①②③④⑥ |
| Men (2009) | 70/70 | 77/63 | C:3-12; A:3-12 | TRQ+Azithromycin vs. Azithromycin | 0.3-0.5mg/(kg·d) | NA | 7-10 | Identical | ① |
| Cheng (2008) | 35/35 | 39/31 | C:0-14; A:0-14 | TRQ+Azithromycin vs. Azithromycin | 0.3-0.5mg/(kg·d) | Abatement of fever, relieving a cough and preventing asthma, nutritional support | 14 | Identical | ①②③④⑥ |
| Li (2007) | 42/40 | 46/36 | C:3-12; A:3-12 | TRQ+Azithromycin vs. Azithromycin | 0.5-1.0mg/(kg·d) | NA | 14 | Identical | ①②③⑥ |
| Xiao (2014) | 39/39 | 40/38 | 0.5-11(5.26±2.75) | XYP+Azithromycin vs. XXN+Azithromycin | 0.2-0.4ml/(kg·d) 0.5mg/(kg·d) | Antiallergic, abatement of fever, relieving a cough and preventing asthma, | 5 | Identical | ①⑤ |
| Duan (2014) | 63/63 | 66/60 | C:5.4±1.1; A:5.4±1.1 | XYP+Azithromycin vs. XXN+Azithromycin | 0.2-0.4ml/(kg·d) 0.5mg/(kg·d) | Abatement of fever, reducing phlegm, relieving a cough and preventing asthma | 7 | Identical | ①②③④ |

N, number; E, experimental; C, control; M, male; F, female; d, day; ①, clinical effective rate; ②, disappearance time of fever; ③, disappearance time of cough; ④, disappearance time of pulmonary rale; ⑤, average hospitalization time; ⑥, disappearance time of pulmonary shadows in X-ray; ⑦, TNF-α; ⑧, IL-6; RDN, Reduning injection; TRQ, Tanreqing injection; XXN, Xixinnao injection; XYP, Xiyanping injection; YHN, Yanhuning injection.

Reference:

Chen Y. Therapeutic effect of azithromycin combined with hot poisoning on mycoplasma pneumonia. Contemporary Medicine. 2015; 40(8):127-128.

Guo H, Ge L. Effect of YanHuNing combined with azithromycin on inflammatory factors and immune function in children with mycoplasma pneumonia. Journal o f H ainan Medical University. 2017; 23(20):2815-2817.

Huang ZN. Changes of Serum Inflammatory Factors in Children with Mycoplasma Pneumoniae Infected with Azithromycin Combined with Redoxnin. Journal of Clinical Medicine. 2017; 37(7):91-93.

Liang CX, Jiang XF, Jiang DH. Clinical efficacy of azithromycin combined with Xiyanping injection in the treatment of mycoplasma pneumonia in children. Drugs and Clinical. 2015(1):46-48.

Wang G. Effect of Yanhuning Treatment on Mycoplasma Pneumonia in Children and Its Effect on Immune Function. China Modern Medicine. 2018(1):101-103.

Wang HY. Therapeutic effect and mechanism of azithromycin combined with Xiyanping on mycoplasma pneumonia in children. Modern Journal of Integrated Traditional Chinese and Western Medicine. 2013; 22(20):2221-2223.

Wang SQ, Shi YN. Effect of heat toning and azithromycin on inflammatory factors and clinical efficacy of children with mycoplasma pneumonia. Guangxi Medical Journal. 2016; 38(8):1078-1080.

Xu DJ, Yang RY, Luo XP, Zhao ZY. Clinical observation of azithromycin combined with hot poisoning injection in the treatment of mycoplasma pneumoniae pneumonia in children. E-Journal of Translational Medicine. 2014(2):28-29.

Yin WP, Xia J. Clinical Observation of 35 Cases of Mycoplasma Pneumonia in Children Treated by Sequential Therapy with α-Ashina Injection. Yunnan Journal of Traditional Chinese Medicine and Materia Medica. 2009; 30(1):39-40.

Yu ZX, Xie XQ. Influence of Combination of Traditional Chinese and Western Medicine on Inflammatory State and Treatment Effect of Children with Mycoplasma Pneumonia. Medical Innovation of China. 2015(34):99-102.

Zhang YX. Therapeutic effect of azithromycin and thermotonin on mycoplasma pneumonia in children. Chinese journal of ethnomedicine and ethnopharmacy. 2013; 22(10):101-101.

Zhu MT, Luo HM. The effect of sequential treatment of azithromycin and thermotonin on mycoplasma pneumonia in children and its influence on serum inflammatory factors. Journal of Bethune Medical Science. 2018(1) :101-103.

Zuo ZX. Clinical Observation on Treatment of Mycoplasma Pneumonia in Children with Xiyanping Injection Combined with Azithromycin. Medical Information. 2015(2):266-266.

An WP, Li R, Guo HM, Xu BX. Clinical Study on Azithromycin Combined with Hot Tonic for Mycoplasmal Pneumonia in Children. China Practical Medicine. 2012; 07(16):28-29.

Bai L. Efficacy of Tanreqing injection combined with azithromycin in treating 60 cases of mycoplasma pneumonia in children. Chinese Pediatrics Of Integrated Traditional And Western Medicine, 2013(5):421-422.

Cao HX. 124 cases of mycoplasma pneumonia in high altitude area treated by Tanreqing Injection. Journal of Emergency in Traditional Chinese Medicine, 2011, 20(1):108-108.

Cao MQ. Observation on the Curative Effect of Azithromycin Combined with YanHuaNing on Mycoplasma Pneumonia in Children. Practical Journal of Cardiac Cerebral Pneumal and Vascular Disease. 2011, 19(4):609-610.

Cao SP. Observation on the Curative Effect of Azithromycin Combined with YanHuaNing on Mycoplasma Pneumonia in Children. Journal of Practical Traditional Chinese Medicine. 2009, 25(7):462-463.

Chen D. Azithromycin combined with Tanreqing Injection in the treatment of mycoplasma pneumonia in children. China Foreign Medical Treatment, 2013, 32(27):111-111.

Chen HH, Chen Z. Treatment of 60 Cases of Mycoplasma Pneumonia with Azithromycin Combined with Redoxing Injection. Herald of Medicine. 2014; 33(1):65-66.

Chen SP, Yang Jing, Song Yan. Yan Hu Ning combined with azithromycin for the treatment of mycoplasma pneumonia in children. Journal of Aerospace Medicine. 2007, 18(1):21-22.

Chen XM, Zhang YP. Clinical Observation on 28 Cases of Mycoplasma Pneumonia in Children Treated with Azithromycin Combined with Xiyanping. Practical Clinical Journal of Integrated Traditional Chinese and Western Medicine. 2013; 13(5):5-6.

Chen ZD. Clinical Observation on Mycoplasma Pneumonia in Children Treated with Azithromycin and Tanreqing. World Latest Medicine Information. 2015, 15(3):86-87.

Cheng AP. Clinical observation of Azithromycin combined with Tanreqing in treatment of mycoplasma pneumonia in children. China Practical Medical, 2012, 7(31):9-10.

Cheng SB. Clinical observation of Tanreqing Injection in adjuvant treatment of mycoplasma pneumonia in children. Chinese Journal of Practical Medicine, 2008, 35(16):82-83.

Cheng ZM, Cao Mei, Ji SB. Efficacy of Azithromycin sequential therapy combined with Tanreqing injection for treatment of children with mycoplasma pneumonia. Journal of Hainan Medical University. 2017, 23(6):789-792.

Cui BZ, Chen XT, Duan MT. Observation on the efficacy of Tanreqing combined with azithromycin in the treatment of mycoplasma pneumonia in children. China Health Care & Nutrition, 2013, 23(7):3879-3880.

Cui RR, Liu XX. Azithromycin combined with YanHuaNing for the treatment of mycoplasma pneumonia in children. Maternal and Child Health Care of China. 2010, 25(16):2305-2306.

Dai YJ. Treatment of 41 Cases of Mycoplasma Pneumonia in Children with Combination of Traditional Chinese and Western Medicine. Acta Chinese Medicine and Pharmacology. 2012; 40(6):94-96.

Deng SQ. Effect of Tanreqing Injection Combined with Azithromycin on Content of C-reactive Protein and Myocardial Enzyme in Mycoplasma Pneumonia in Children. Modern Journal of Integrated Traditional Chinese and Western Medicine. 2015, 24(20):2226-2228.

Deng Y, Li L. Clinical Observation of Xiyanping Injection Combined with Azithromycin Sequential Therapy for Children with Mycoplasma Pneumonia. Hebei Medicine. 2015(10):1613-1616.

Dong YW. Experience in the diagnosis and treatment of 80 cases of mycoplasma pneumonia in children. Journal of Frontiers of Medicine. 2014; (32):152-152.

Dou YF, Shi YP, Lei W, Li D. Clinical Observation on 40 Cases of Child Mycoplasma Pneumonia Treated by Xiyanping. Shaanxi Medical Journal. 2016; 45(10):1420-1421.

Du HR, Wang YM. Clinical Observation on Mycoplasma Pneumonia in Children Treated with Azithromycin Sequential Therapy Combined with Redoxing Injection. Hebei Medical. 2011; 33(19):2944-2945.

Du JH. Efficacy of Azithromycin combined with Tanreqing in treatment of mycoplasma pneumonia in children. Chinese Journal of Practical Medicine, 2011, 38(20):96-97.

Du YY, Yang GM, Li SH. Clinical Study of Xiyanping Combined with Azithromycin Sequential Therapy for Mycoplasma Pneumonia in Children. Journal of China Traditional Chinese Medicine Information. 2011; 03(8):174-174.

Duan QB. Comparison of Effects of Different Drug Combination Regimen in the Treatment of Mycoplasma Pneumonia in Children. Medical Information, 2014, 33(36):112-112.

Duan XZ, Feng XC. Clinical Observation on Mycoplasma Pneumoniae Pneumonia in Children Treated with Repontocine Injection. Journal of Changchun University of Traditional Chinese Medicine. 2011; 27(2):171-172.

Fan GQ. Azithromycin combined with Tanreqing in the treatment of mycoplasma pneumonia in children. Medical Information, 2011, 24(23):713-713.

Fan TL, Xia YH. Efficacy and Safety Evaluation of Azithromycin Combined with Hot Tonic for Mycoplasma Pneumonia in Children. Journal of North Pharmacy. 2016; 13(11):137-138.

Fang Lei, Zhou HR, Chen Hua. Observation on Curative Effect of Tanreqing Injection Combined with Azithromycin on Mycoplasma Pneumonia in Children. Journal of New Chinese Medicine. 2015, 47(12):158-160.

Feng YJ, Sun XM. Observation of Curative Effect of Yanhuening Treatment of Mycoplasma Pneumoniae Pneumonia in Children. Journal of Community Medicine. 2011, 09(7):21-22.

Fu JX. Clinical observation of Tanreqing combined with azithromycin in the treatment of mycoplasma pneumonia in children. Chinese Journal of Clinical Rational Drug Use. 2014(19):59-59.

Gao XQ. Experience in the Joint Application of Retinol and Azithromycin in Treatment of Pneumonia. Journal of Aerospace Medicine. 2015; 26(2):219-219.

Guan QH. Clinical Observation on Treatment of Mycoplasma Pneumonia in Children with Xiyanping Injection Combined with Azithromycin. Journal of Frontiers of Medicine. 2016; 6(27):117-118.

Guo JZ. Clinical observation on the efficacy of azithromycin combined with Tanreqing in the treatment of mycoplasma pneumonia in children. Shanxi Medical Journal. 2016, 45(7):810-811.

Han XD. Clinical Analysis of Azithromycin Combined with Tanreqing in Treating Mycoplasma Pneumoniae Pneumonia in Children. World Latest Medicine Information. 2016, 16(51).115-142.

He SQ, Wen XL, Xu N. Clinical research of Tanreqing joint Azithromycin sequential therapy in children with mycoplasma pneumoniae pneumonia. China Medical Herald, 2009, 6(35):49-50.

Hou JJ. Therapeutic Efficacy and Safety Evaluation of Retinol Injection Combined with Azithromycin Sequential Therapy for Mycoplasma Pneumoniae Pneumonia in Children. Journal of Aerospace Medicine. 2017; 28(6):726-728.

Hu B. Efficacy of Tanreqing in adjuvant treatment of mycoplasma pneumonia in children. China Practical Medical.2014(27):192-193.

Hu XJ. Efficacy on Mycoplasma Pneumonia Treated with Azithromycin and Tan-reqing Injection in Children. World Journal of Integrated Traditional and Western Medicine, 2014(5):514-516.

Hu YJ. 70 Cases of Infantile Pneumonia Mycoplasma Pneumonia Treatment Effect. China Health Standard Management. 2015, 6(1):70-71.

Huang YM. Treatment of Mycoplasma Pneumoniae Pneumonia in Children with Thermotonine Injection and Azithromycin Sequential Therapy. The Journal of Medical Theory and Practice. 2015(3):336-337.

Huang ZW. Clinical observation of intravenous azithromycin combined with Tanreqing injection in treatment of children with mycoplasma pneumoniae pneumonia. Chinese Journal of New Clinical Medicine, 2014, 7(10):953-956.

Huo CJ. Treatment of 40 Cases of Mycoplasma Pneumoniae Pneumonia in Children. China Health Care & Nutrition. 2013(11) :429-430.

Jia HQ. Clinical observation of azithromycin combined with Tanreqing in the treatment of mycoplasma pneumoniae pneumonia in children. The Medical Forum. 2016, 20(9):1223-1224.

Jiang Lei. Observation on the Curative Effect of Azithromycin Combined with YanHuaNing on Mycoplasma Pneumonia in Children. Chinese Journal of Modern Drug Application. 2010, 04(17):145-145.

Jiang S. Combination of azithromycin and Xiyanping injection in the treatment of 44 children with mycoplasma pneumonia. Clinical Research. 2013(s1):119-120.

Jiang YP. Clinical Observation on 132 Cases of Mycoplasma Pneumonia in Children Treated by Yanhuening. Medical Innovation of China. 2008, 5(33):14-15.

Lan B. Clinical analysis of azithromycin combined with and rographolide in treating children mycoplasmal pneumonia. Tibetan Medicine. 2016; 6(1):1-3.

Li FR, Zhou DX, Wang YQ, Fan SJ, Guo LL. Clinical Analysis of Mycoplasma Pneumonia in Children Treated by Yanhuning and Azithromycin. Chinese Journal of Modern Drug Application. 2010, 4(4):112-113.

Li HQ. Therapeutic effect of sequential treatment of Xiyanping injection combined with azithromycin on mycoplasma pneumonia in children. Mod Diagn Treat. 2015; 26(21):4833-4834.

Li JJ. Clinical observation of azithromycin combined with Xiyanping in the treatment of mycoplasma pneumonia in children. China Health Care & Nutrition. 2017; 27(23):200-201.

Li LX. Curative effect observation of Tanreqing combined with Azithromycin in the treatment of mycoplasma pneumoniae pneumonia in children. China Modern Medicine, 2012, 19(11):71-72.

Li Qin. Clinical efficacy of Tanrenqing injection on combined with azithromycin in treating pediatric patients with MPP and safety evaluation. Health Care Guide. 2017(7):3-4.

Li WH Observation of curative effect by azithromycin combined with Tanreqing in the treatment of 64 pediatric mycoplasma pneumoniae pneumonia cases. Chinese Journal of Modern Drug Application. 2015, 9(23):1-2.

Li XJ. Clinical Analysis of 80 Cases of Mycoplasma Pneumonia in Children Treated by Yanhuning and Azithromycin. Journal of Frontiers of Medicine. 2015; 5(36).120-121.

Li XY. Effect of Tanreqing Injection on mycoplasma pneumonia in children. Chinese Community Doctors, 2007(12):90-90.

Li YH. Analysis of clinical efficacy of azithromycin combined with YanHuaNing in the treatment of mycoplasma pneumonia in children. World Latest Medicine Information. 2017(49).107-111.

Li YJ, Zhou XH. Therapeutic effect of Xiyanping combined with azithromycin on mycoplasma pneumonia in children. Journal of Clinical Medical. 2015(16):3199-3199.

Li YL. Clinical Study on Pediatric Mycoplasma Pneumonia Treated by Tanreqing Combined with Azithromycin. Henan Traditional Chinese Medicine. 2015, 35(10):2534-2536.

Li YN, Xu DH, Dai KK. Therapeutic Effect of Repontophane Injection Combined with Azithromycin for Mycoplasma Pneumoniae Pneumonia in Children. China Medicine. 2014; 9(6):894-896.

Liao PL, Chen W, Feng YP, Wan DH. Clinical observation of Tanreqing Injection in treating 82 cases of mycoplasma pneumonia in children. The Journal of Medical Theory and Practice, 2011, 24(7):787-788.

Lin Feng. Clinical Observation on Treatment of Mycoplasma Pneumonia in Infants and Young Children with Azithromycin and Tanreqing. Journal of Frontiers of Medicine. 2017, 7(20):165-166.

Lin Jian, Xu ZY, Xing DW. Efficacy of Tanreqing Injection Combined with Azithromycin in Treatment of Mycoplasma Pneumonia in Children and Its Effects on Inflammatory Factors. Chinese Archives of Traditional Chinese Medicine. 2017, 35(9):2418-2420.

Lin YP. Therapeutic Analysis and Safety Evaluation of Mycoplasma Pneumonia in Children Treated by Integrative Chinese and Western Medicine. Journal of Frontiers of Medicine. 2015(11):110-110.

Liu JP. Clinical efficacy of Azithromycin and Tanreqing in treatment of children with mycoplasma pneumonia. China Modern Doctor, 2013, 51(32):80-82.

Liu Juan. Clinical observation of azithromycin combined with Tanreqing in the treatment of mycoplasma pneumoniae pneumonia in children. Journal of Frontiers of Medicine. 2016, 6(10):167-168.

Liu JY. Observation of Curative Effect of Azithromycin Combined with Tanreqing Injection on Mycoplasma Pneumoniae Pneumonia in Children. China Medical Engineering. 2016; 24(6):45-46.

Liu P. Azithromycin combined with Tanreqing in the treatment of mycoplasma pneumonia in children. Chinese Journal of Clinical Rational Drug Use,2014,7(5):28.

Liu XY, Tao Yuan. Efficacy of Combination of Yanhuning and Azithromycin in Treatment of Mycoplasma Pneumonia in Children ：An Observation of 48 cases. China Modern Doctor. 2011, 49(20):71-72.

Liu YL, Gou CF. Evaluation of the effect of azithromycin and yanhuning for the treatment of mycoplasma pneumonia in children. Psychologist. 2017; 23(12): 81-82.

Liu YM. Clinical Observation of 88 Cases of Mycoplasma Pneumonia in Children Treated with Azithromycin Combined with Hot Tonic. China Health Industry. 2011(20):81-81.

Liu ZY. Clinical study on Tanreqing Injection combined with azithromycin in sequential treatment of children with mycoplasma pneumonia. Drugs & Clinic. 2017, 32(2):237-240.

Lu GH. Efficacy of Azithromycin combined with Tanreqing in treatment of mycoplasma pneumonia in children. Clinical Medicine, 2014,34(5):92-93.

Lu HL. Observation of Curative Effect of Hypertonin Combined with Azithromycin on Mycoplasma Pneumonia in Children. Modern Preventive Medicine. 2008; 35(15):3007-3007.

Lu JL. Clinical Observation of Azithromycin Combined with YanHuaNing in the Treatment of Mycoplasma Pneumonia in Children. Chinese Journal of Modern Drug Application. 2011, 05(1):112-113.

Luo AH. Clinical observation of Yanhuening treatment of children with mycoplasma pneumonia pneumonia. Asia-Pacific Traditional Medicine. 2010, 6(5):83-84.

Ma SJ. Therapeutic Efficacy of Yanhuning and Azithromycin in the Treatment of Mycoplasma Pneumonia in Children. The Medical Journal of the Present Clinical. 2015; (1):1210-1210.

Ma ZL. Azithromycin combined with Tanreqing Injection in the treatment of mycoplasma pneumonia in children. Chinese Community Doctors, 2013, 15(7):209-210.

Mei Y, Xue LX. Clinical study of Azithromycin combined with Tanreqing in treatment of mycoplasma pneumonia in children. China Health Care & Nutrition, 2014(1):441-442.

Men ZY, Qi XZ, Zang ZY. Treatment of 70 cases of mycoplasma pneumonia in children with integrated traditional Chinese and Western Medicine. Modern Traditional Chinese Medicine, 2009, 29(1):6-7.

Meng HX, Liu JJ. Clinical Observation of Mycoplasma Pneumonia Treated by Yanhuning and Azithromycin. Clinical Focus. 2010, 25(3):250-251.

Ning W. Effect of Xiyanping Combined with Azithromycin on Mycoplasma Pneumonia in Children. Chinese and Foreign Medical Research. 2014(35):146-147.

Qi Bing Efficacy on mycoplasma pneumonia treated with Tanreqing injection and Azithromycin in 43 cases of children. Journal of Medical Forum. 2016, 37(10):60-61.

Shang B, Lin ZS. Azithromycin combined with Tanreqing Injection in the treatment of Mycoplasma pneumoniae in children. Journal of Qiqihar Medical College, 2009, 30(6):689-690.

Shen B. Treatment of Mycoplasma Pneumoniae Pneumonia in Children with Heat Tonicing Injection. Journal of Guide of China Medicine. 2017; 15(5):188-189.

Sheng AM. Tanreqing injection combined with azithromycin in the treatment of mycoplasma pneumonia in children. Chinese Journal of Practical Medicine, 2014, 30(12):117-118.

Song W. Clinical efficacy of azithromycin combined with hot poisoning injection in the treatment of mycoplasma pneumonia. Jiangsu Medical Journal. 2011; 37(4):489-490.

Song XP. Therapeutic effect of Yanhuning and azithromycin on mycoplasma pneumonia in children. Medical Innovation of China. 2011, 08(18):76-77.

Song YF. Treatment of Mycoplasma Pneumoniae Pneumonia in Children by Integrative Chinese and Western Medicine: A Clinical Observation of 40 Cases. Chinese Pediatrics of Integrated Traditional and Western Medicine. 2009; 1(4):364-365.

Song YH, Yang YH. Observation of Curative Effect of α-Asarone for Children Mycoplasma Pneumonia. Chinese Traditional Patent Medicine. 2005; 27(5):554-555.

Su Wei. Clinical Observation on Mycoplasmal Pneumonia in Children Treated by Yanhuning and Azithromycin. Modern Journal of Integrated Traditional Chinese and Western Medicine. 2011, 20(5):542-543.

Sun GB. Clinical observation of Xiyanping combined with azithromycin in the treatment of mycoplasma pneumonia in children. China Practical Medicine. 2012; 07(27):148-149.

Tao BT. Therapeutic effect of pyronine combined with azithromycin on mycoplasma pneumonia in children and its influence on serum inflammatory factors. Journal of North Pharmacy. 2017; 14(1):98-99.

Wang AM, Han Y, Li H, Chen HT. Efficacy of Tanreqing Injection in adjuvant treatment of mycoplasma pneumonia in children. China Pharmacist, 2013, 16(2):263-265.

Wang CX, Liu C. Treatment of 40 Cases of Mycoplasma Pneumonia in Children with Hot Tonicing Injection. Medical Information. 2014(19):76-76.

Wang H. Treatment of 52 children with mycoplasma pneumonia with azithromycin combined with hot poisoning. Journal of Health For Everyone. 2016(23):98-99.

Wang HJ. Clinical observation of Azithromycin combined with Tanreqing in treatment of mycoplasma pneumonia in children. Jilin Medical Journal, 2012, 33(25):5464-5465

Wang HJ. Clinical observation of Azithromycin combined with Tanreqing in treatment of mycoplasma pneumonia in children. Jilin Medical Journal, 2012, 33(25):5464-5465

Wang JX, Zhou YL, Li J. Observation on the efficacy of Tanreqing combined with azithromycin in the treatment of mycoplasma pneumonia in children. Modern Journal of Integrated Traditional Chinese and Western Medicine, 2013, 22(28):3119-3121.

Wang L, Wu CQ. The Effect of Tanreqing Injection and Azithromycin in the Treatment of Children with Mycoplasma Pneumonia Pneumoniae. Journal of Pediatric Pharmacy, 2013(10):28-30.

Wang PH. Clinical analysis of mycoplasma pneumonia in children. Yiayao Qianyan, 2013(36):192-193.

Wang W. The application value of azithromycin combined with Xiyanping in the treatment of mycoplasma pneumonia in children. China Continuing Medical Education. 2016; 8(11):222-223.

Wang XL. Therapeutic Efficacy of Retoxin Injection Combined with Azithromycin for Mycoplasma Pneumonia in Children. Chinese Journal of Modern Drug Application. 2014(5):135-136.

Wang XY. Therapeutic effect of Yanhuning combined with azithromycin on mycoplasma pneumonia in children. Chinese Journal of Primary Medicine and Pharmacy. 2012, 19(21):3318-3319.

Wang YP, Yu WZ, Liu XM. Evaluation of the value of azithromycin combined with Tanreqing in the treatment of mycoplasma pneumonia in children. Journal of Taishan Medical College. 2016, 37(12):1407-1408.

Wang ZJ. Observation of Tanreqing combined with azithromycin in treatment of mycoplasma pneumonia in children. Medical Information, 2011, 24(5):2059-2060.

Wei Y. Treatment of 30 Cases of Mycoplasma Pneumonia in Children with Repontocin Injection. Nei Mongol Journal of Traditional Chinese Medicine. 2013; 32(8):30-31.

Wei YS. Clinical observation of Azithromycin combined with Tanreqing in treatment of mycoplasma pneumonia in children. China Medical Engineering, 2013(6):64-64.

Wen Hong. Efficacy of Azithromycin Combined with Ｔanreqing Inｊection in Ｔreatment of Mycoplasma Pneumonia in Children and Its effect on Serum CRP and Immunoglobulin Levels in 40 Cases. China Pharmaceuticals. 2014, 23(19):83-84.

Wen RQ. Clinical Observation on 40 Cases of Children with Mycoplasma Pneumonia Treated by Yanhuning and Azithromycin. Guiding Journal of Traditional Chinese Medicine and Pharmacy. 2014; 20(8):115-116.

Wu JP. Observation of Curative Effect of Azithromycin Combined with Tanreqing on Mycoplasma Pneumonia in Children. Journal of Frontiers of Medicine. 2017, 7(32):32-33.

Wu XF, Bai DB, Wang BY, Chen RY, Ren J. X-ray characteristics and curative effect analysis of 80 children with Mycoplasma pneumoniae pneumonia. Journal of Clinical Pulmonary Medicine. 2014(9):1714-1716.

Wu YZ, Zhang Y. Therapeutic Effect of Reminol Injection Combined with Azithromycin in Treating Pediatric Pneumonia with Bronchial Pneumonia. Strait Pharmaceutical Journal. 2013; 25(12):143-145.

Xiao WH, Peng XJ. Effect observation of xiyanping and asarone respectively combined with azithromycin treating Mycoplasma pneumonia in children. China Modern Medicine, 2014, 21(2):83-84.

Xiao WH. Clinical observation of Tanreqing combined with azithromycin in the treatment of mycoplasma pneumonia in children. Journal of Guiyang College of Traditional Chinese Medicine, 2011, 33(6):74-75.

Xing, XH. Clinical observation of Azithromycin combined with Tanreqing in treatment of mycoplasma pneumonia in children. Medical Information, 2014(1):324-325.

Xu DY. Comparative analysis of Tanreqing combined with azithromycin in treatment of mycoplasma pneumonia in children. Chinese Journal of Prevention and Control of Chronic Diseases, 2011, 19(6):631-632.

Xu QM. Treatment of 20 Cases of Mycoplasma Pneumonia in Children with Retinol Injection Combined with Azithromycin. Chinese journal of ethnomedicine and ethnopharmacy. 2011; 20(24):114-115.

Yan Hong, Wan YF, Chen XJ. Treatment of 40 Cases of Pediatric Mycoplasma Pneumonia with Combination of Traditional Chinese and Western Medicine. Chinese Medicine Modern Distance Education of China. 2012, 10(4):149-149.

Yan J, Yi JB, Xiao XH. Observation of the Curative Effect of Azithromycin Combined with Tanreqing in treating Child with Mycoplasma Pneumoniae Pneumonia. Progress in Modern Biomedicine, 2010, 10(12):2339-2341.

Yang J. Clinical evaluation of Xiyanping injection combined with azithromycin in the treatment of children with mycoplasma pneumonia. Chinese medicine. 2014; 9(12):1746-1748.

Yang J. Treatment of Mycoplasma Pneumoniae Pneumonia in Children with Thermotonine Injection and Azithromycin Sequential Therapy. Journal of Acta Chinese Medicine and Pharmacology. 2015(4):101-103.

Yang XX. Analysis of Clinical Efficacy of Yan Hu Ning Injection in the Treatment of Mycoplasma Pneumonia in Children. World Latest Medicine Information. 2015; 15(60).50-61.

Yang YR, He W. Observation on curative effects of azithromycin combined with dehydroandrographolide succinate on children with mycoplasma pneumonia. Medical Journal of National Defending Forces in Southwest China. 2016; 26(4):417-419.

Yao H. Azithromycin Sequential Therapy Combined with Tanreqing Injection in the treatment of mycoplasma pneumonia in children. Jin Ri Jian Kang, 2014(6):81-81

Yao L. Asarum injection for the treatment of mycoplasma pneumonia in children. China Modern Medicine. 2011; 18(16):66-67.

Ye JY. Clinical Analysis of Mycoplasma Pneumonia in Children Treated by Yanhuning and Azithromycin. Journal of Mathematical Medicine. 2015; 22(5):728-729.

Yi YQ. Treatment of 32 Children with Mycoplasma Pneumoniae Pneumonia with Tanreqing Injection Combined with Azithromycin. Medical Information. 2015, 28(18):296-296.

Yong QJ, Yin TJ, Chen ZY, Wu Y, Cheng S. Detection and Mechanism Analysis of Serum Inflammatory Factors Associated with Mycoplasma Pneumonia in Children. China &Foreign Medical Treatment. 2017; 36(15):143-145.

Yu GX. Clinical Observation on Pediatric Pneumonia Pneumonia Treated with Tanreqing Injection. Chinese Journal of Modern Drug Application. 2016, 10(2):183-184.

Yu YJ. Effect of Yanhuning and Azithromycin on Mycoplasma Pneumonia in Children. Diet and health. 2017; 4(23).54-55.

Yuan CF. Analysis of Clinical Diagnosis and Treatment of Mycoplasma Pneumoniae Pneumonia in Children. Chinese and Foreign Medical Research. 2015, 25(14):97-98.

Yuan YF, Liu QG, Sun LQ. Efficacy of Azithromycin combined with Tanreqing in treatment of mycoplasma pneumonia in children. Chinese Journal Of Modern Drug Application, 2013, 7(14):133-134.

Zhan HZ, Yan HX. Clinical observation of Tanreqing injection combined with azithromycin as sequential therapy for children with mycoplasma pneumonia. Journal of Clinical Pulmonary Medicine, 2013, 18(8):1435-1436.

Zhang HN. Evaluation of the efficacy of azithromycin combined with Tanreqing in the treatment of mycoplasma pneumonia in children. Journal of China Prescription Drug. 2016; 14(2):72-73.

Zhang HX. Clinical Effect of Tanreqing Combined with Azithromycin Sequential Therapy in Children with Mycoplasma Pneumonia. Medical Equipment. 2017, 30(11):11-12.

Zhang Jie. Effect of Yanhuning on Serum TNF-α and IL-6 in Patients with Mycoplasma Pneumonia in Children. Journal of Clinical Pulmonary Medicine. 2014; 19(12):2283-2284.

Zhang JW. Effect of Retinol Injection on Lymphocyte Subsets and Serum Th17, IL-17A in Children with Mycoplasma Pneumoniae Pneumonia. Journal of Medical Forum. 2017(7):47-49.

Zhang Li, Liang SJ. Observation on the Curative Effect of Combined Chinese and Western Medicine on Mycoplasma Pneumonia in Children. Contemporary Medical Symposium. 2012; 10(8):461-462.

Zhang Li. Therapeutic effect of Yanhuning combined with azithromycin on mycoplasma pneumonia in children. Contemporary Medical Symposium. 2014; 12(15):218-220.

Zhang Q. Azithromycin combined with Tanreqing in the treatment of mycoplasma pneumonia in children. Public Medical Forum Magazine, 2011, 15(34):1113-1114.

Zhang Xia, Shi ZX. Clinical analysis of azithromycin combined with Yanhuning in treatmentof Mycoplasma pneumonia in children. China Modern Medicine. 2014; 21(5):73-74.

Zhang XX, Guo Q. Treatment of Mycoplasma Pneumonia with Azithromycin Treated with Xiyanping. Second Summit Forum for the Exchange of Experiences in Clinical Acute and Severe Diseases. 2015

Zhang XY. The clinical efficacy of Tanreqing Injection combined with Azithromycin sequential therapy in children with mycoplasma pneumonia. China Medical Herald, 2011, 08(28):56-57.

Zhang YC. Analysis of Effect of Azithromycin Combined with Asarone Injection on Mycoplasma Pneumonia in Children. Henan Medical Research. 2014; 10(22):52-53.

Zhang YM. Clinical Observation on Treatment of Mycoplasma Pneumoniae Pneumonia in Children with Tanreqing Injection Combined with Azithromycin. Modern Diagnosis and Treatment. 2016, 27(17):3172-3174.

Zhang ZW. Observation of clinical treatment of mycoplasma pneumonia in children. China rural health, 2012(z2):116-117.

Zhao H, Huang GJ. Therapeutic Efficacy of Retinol Injection in Children with Mycoplasma Pneumoniae Pneumonia and Its Effect on Inflammatory Cytokines. Journal of Frontiers of Medicine. 2018; 8(2): 89-90.

Zhao PA. Combined use of azithromycin and Tanreqing for the treatment of mycoplasma pneumonia in children. Contemporary Medical Symposium. 2016, 14(6):128-129.

Zheng HZ. Clinical observation of Azithromycin combined with Tanreqing in treatment of mycoplasma pneumonia in children. China Foreign Medical Treatment, 2014, 7(9):963-964.

Zhong XM, Deng Yan, Chu XM. Effect of Yanhuning on serum inflammatory cytokines and immunological function of children with my coplasma pneumonia. Journal of Hainan Medical University. 2015; 21(9):1262-1264.

Zhou JX. Analysis of the effect of azithromycin combined with Tanreqing on children with mycoplasma pneumonia pneumonia. Chinese and Foreign Medical Research. 2017, 15(24):136-137.

Zhou M. Clinical Observation of Azithromycin Sequential Therapy Combined with Hot Tonic for Mycoplasma Pneumonia in Children. Medicine and Health. 2015(7):151-152.

Zhou Qing. Azithromycin combined with potassium sodium dehydroandroan drographolide succinate versus azithromyein alone for mycoplasma pneumonia in children. International Medicine and Health Guidance News. 2012; 18(23):3427-3429.

Zi HF, Li PB. Tanreqing combined with azithromycin in the treatment of 53 cases of mycoplasma pneumonia in children. Chinese Journal of Coal Industry Medicine, 2012, 15(5):693-694.
